# Brain insulin action on peripheral insulin sensitivity in women depends on menstrual cycle phase

**DOI:** 10.1038/s42255-023-00869-w

**Published:** 2023-09-21

**Authors:** Julia Hummel, Charlotte Benkendorff, Louise Fritsche, Katsiaryna Prystupa, Andreas Vosseler, Sofiya Gancheva, Sandra Trenkamp, Andreas L. Birkenfeld, Hubert Preissl, Michael Roden, Hans-Ulrich Häring, Andreas Fritsche, Andreas Peter, Robert Wagner, Stephanie Kullmann, Martin Heni

**Affiliations:** 1 Institute for Diabetes Research and Metabolic Diseases of the Helmholtz Center Munich at the University of Tübingen, Eberhard Karls University Tübingen, Tübingen, Germany; 2 Department of Internal Medicine I, Division of Endocrinology and Diabetology, University of Ulm, Ulm, Germany; 3 Department of Internal Medicine, Division of Diabetology, Endocrinology and Nephrology, Eberhard Karls University Tübingen, Tübingen, Germany; 4 German Center for Diabetes Research (DZD), München-Neuherberg, Germany; 5 Institute for Clinical Diabetology, German Diabetes Center, Leibniz Institute for Diabetes Research at Heinrich Heine University Düsseldorf, Düsseldorf, Germany; 6 Department of Endocrinology and Diabetology, University Hospital Düsseldorf, Heinrich Heine University Düsseldorf, Düsseldorf, Germany; 7 Department for Diagnostic Laboratory Medicine, Institute for Clinical Chemistry and Pathobiochemistry, Eberhard Karls University Tübingen, Tübingen, Germany

**Keywords:** Endocrine system and metabolic diseases, Endocrine system and metabolic diseases, Neuroendocrinology

## Abstract

Insulin action in the human brain modulates eating behaviour, whole-body metabolism and body fat distribution^1,2^. In particular, brain insulin action increases whole-body insulin sensitivity, but these studies were mainly performed in lean men^3,4^. Here we investigate metabolic and hypothalamic effects of brain insulin action in women with a focus on the impact of menstrual cycle (ClinicalTrials.gov registration: NCT03929419).

Eleven women underwent four hyperinsulinemic–euglycemic clamps, two in the follicular phase and two in the luteal phase. Brain insulin action was introduced using nasal insulin spray^5–7^ and compared to placebo spray in a fourfold crossover design with change in glucose infusion rate as the primary endpoint. Here we show that during the follicular phase, more glucose has to be infused after administration of nasal insulin than after administration of placebo. This remains significant after adjustment for blood glucose and insulin. During the luteal phase, no significant influence of brain insulin action on glucose infusion rate is detected after adjustment for blood glucose and insulin (secondary endpoint). In 15 other women, hypothalamic insulin sensitivity was assessed in a within-subject design by functional magnetic resonance imaging with intranasal insulin administration^8^. Hypothalamus responsivity is influenced by insulin in the follicular phase but not the luteal phase.

Our study therefore highlights that brain insulin action improves peripheral insulin sensitivity also in women but only during the follicular phase. Thus, brain insulin resistance could contribute to whole-body insulin resistance in the luteal phase of the menstrual cycle.

## Abstract (web summary)

In young women, brain insulin action enhances peripheral insulin sensitivity, but only during the follicular phase of the menstrual cycle. This effect is absent in the luteal phase, possibly due to hypothalamic insulin resistance.

## Main

Over the last decade, the brain was characterized as an insulin-sensitive organ^1^. Insulin crosses the blood–brain barrier^9^ and acts in specialized neurons and glia cells^1^. In humans, insulin regulates brain activity in specific regions, including the hypothalamus^10^, which in turn influences outflows toward the periphery^2^ and modulates food intake and whole-body metabolism^2^. Studies performed in healthy men found that insulin action in the brain suppresses endogenous glucose production^3,11^ and stimulates glucose uptake into peripheral tissues^3^. However, this may require postprandial metabolic conditions^12,13^, as nasal insulin did not modulate endogenous glucose production under systemic fasting insulin concentrations in most^12,14^, but not all, clinical trials^11^.

Nasal insulin administration has been repeatedly used to study insulin action in the human brain^15^. Using this delivery route, substantial amounts of insulin are delivered into the brain^5^, while only a tiny fraction reaches the bloodstream^6^. Thus, nasal insulin allows selective stimulation of brain insulin action without major peripheral side effects. Using this approach, studies have addressed brain-derived modulation of peripheral metabolism in men with normal weight and obesity (for review, see Kullmann et al.^2^).

Initial studies on this topic have already revealed that a substantial number of individuals have a reduced or even absent response to insulin in the brain^16^, a phenomenon termed brain insulin resistance^17^. In affected individuals, brain insulin cannot sufficiently modulate peripheral metabolism^2^. These individuals are predisposed for subsequent weight gain and visceral fat accumulation^18^. The most prominent phenotype linked to human brain insulin resistance is obesity^1^, although other factors, such as genetic background^19^, elevated circulating free fatty acids^20^ and impaired insulin transport across the blood–brain barrier^21,22^, have also been implicated. The detailed regulatory mechanisms for brain insulin resistance, however, are still largely unknown.

Of note, experimental studies suggest considerable sex differences in brain insulin sensitivity. Intracerebroventricular injection of insulin markedly reduced food intake in male rodents, whereas feeding was unaffected in female rodents^23^. In line with these findings, intranasal insulin delivery to the human brain reduced food intake in lean men but not in women^24^. However, some studies reported effects of brain insulin on postprandial snacking in women^25^. Nevertheless, daily nasal insulin administration over 8 weeks reduced body weight and body fat content only in men, whereas women showed no reduction in body adiposity^26^.

To date, no study has investigated sex differences in the regulation of whole-body metabolism by brain insulin action. Because most previous experiments were conducted in healthy young men, here, we aimed to investigate the influence of brain insulin administration on regional brain activity and whole-body glucose metabolism in young women. Because sex hormones are suspected to have a regulatory impact on brain insulin action, we studied naturally cycling women during both the follicular and luteal phases of the menstrual cycle.

## Hyperinsulinemic–euglycemic clamp study

**Fig. 1:**
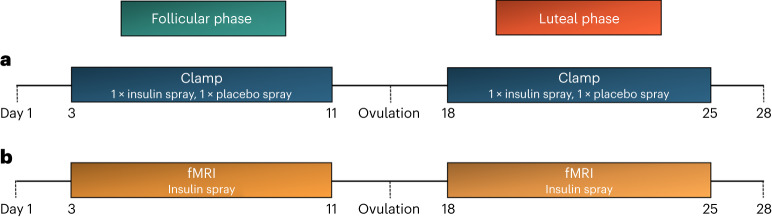
Study outline. **a**,**b**, Timeframes of the experiments in the follicular and luteal phases of the menstrual cycle for the clamp study (**a**) and the fMRI study (**b**). See also CONSORT diagrams in Extended Data Figs. 3 and 4 for the detailed study flow.

For the hyperinsulinemic–euglycemic clamp study, we examined 11 healthy naturally cycling women twice during the follicular phase and twice during the luteal phase (Fig. 1a). Although their body weight and body fat content were not different between phases (both *P* ≥ 0.3), there were differences in sex hormones that ensured our cycle phase determination (Supplementary Table 1). Progesterone and 17-OH-progesterone were higher in the luteal phase, while follicle-stimulating hormone (FSH) was lower. Luteinizing hormone (LH) and estradiol concentrations were not significantly different between phases. The androgens dehydroepiandrosterone (DHEA)-sulfate and androstenedione were higher in the luteal phase, but there were no differences in total and calculated free testosterone. The concentrations of prolactin, anti-Müllerian hormone and morning cortisol were comparable between cycle phases.

Neither the follicular nor the luteal phase showed differences in sex hormone concentrations between the insulin and placebo spray days (all *P* ≥ 0.07).

**Extended Data Fig. 1:**
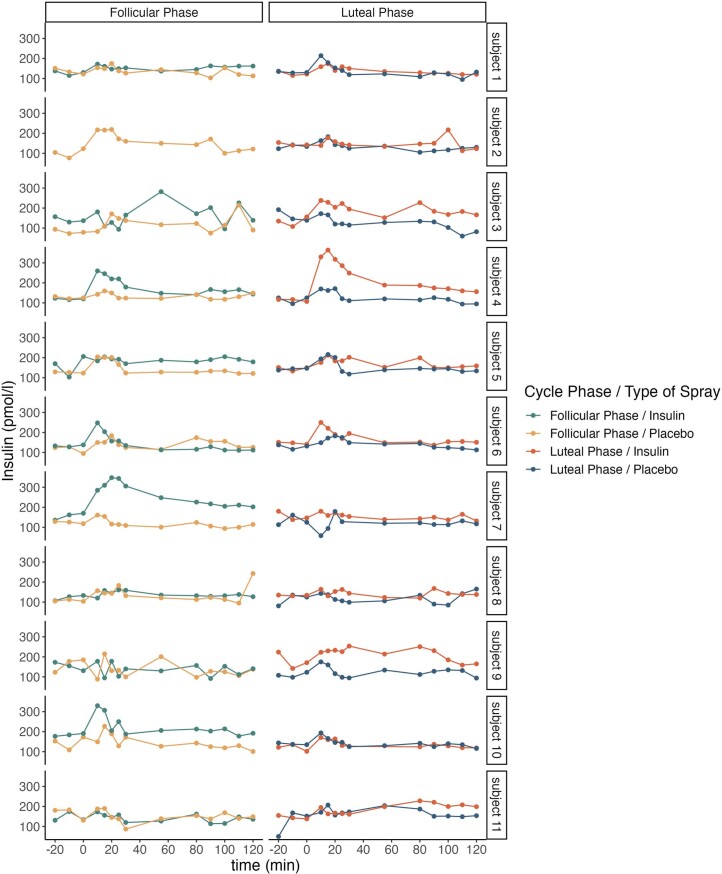
Results from the hyperinsulinemic-euglycemic clamp study – individual courses of serum insulin around nasal insulin administration / mimicking by iv insulin bolus. Presented are the individual courses of serum insulin during the clamp after intranasal insulin spray (green and orange circles) vs placebo spray combined with simultaneous iv insulin bolus (yellow and blue circles) in both phases of the menstrual cycle. Presented are individual data points; N = 11 participants except for the follicular phase where N = 10 for the insulin spray.

Fasting insulin concentrations were comparable between study days (*F*_3,39_ = 1.78, *P*_ANOVA_ = 0.3). After initiation of the hyperinsulinemic–euglycemic clamp, insulin concentrations were also comparable before spray administration (135 ± 20 pmol liter^–1^; *F*_3,39_ = 1.93, *P*_ANOVA_ = 0.1; Extended Data Fig. 1). After administration of insulin nasal spray, there was an increase in circulating insulin concentrations by 65 ± 58 pmol liter^–1^ that peaked after 10 min and was mimicked on placebo days by an intravenous (i.v.) insulin bolus (achieved increase in serum insulin by 30 ± 45 pmol liter^–1^ with peak after 10 min). This increase in serum insulin in the first 10 min as well as in the 20 min after spray administration was comparable between study days (*F*_3,39_ = 1.54 and *P*_ANOVA_ = 0.2 and *F*_3,39_ = 0.86 and *P*_ANOVA_ = 0.5, respectively; Extended Data Fig. 1). Thirty minutes after spray administration, serum insulin concentrations were again in the range of what was measured before spray application on all study days. Also, at the end of the hyperinsulinemic–euglycemic clamp, insulin concentrations were comparable between study days (*F*_3,39_ = 2.43, *P*_ANOVA_ = 0.1; Extended Data Fig. 1).

**Extended Data Fig. 2:**
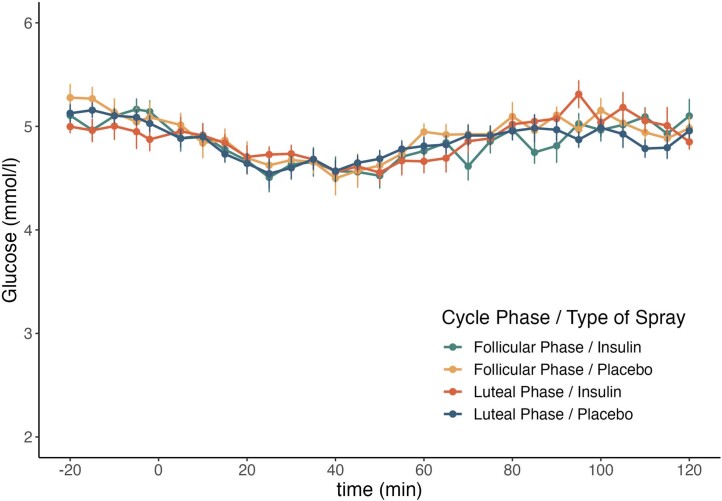
Results from the hyperinsulinemic-euglycemic clamp study – individual courses of plasma glucose around nasal insulin administration / mimicking by iv insulin bolus. Presented are the individual courses of plasma glucose during the clamp after intranasal insulin spray (green and orange circles) vs placebo spray combined with simultaneous iv insulin bolus (yellow and blue circles) in both phases of the menstrual cycle. Presented is a dot plot with means; error bars represent SEM; N = 11 participants except for the follicular phase where N = 10 for the insulin spray.

Plasma glucose concentrations were within the target range and comparable on all study days before spray administration (*F*_3,39_ = 1.03, *P*_ANOVA_ = 0.4; Extended Data Fig. 2) in the 30 min following spray administration (*F*_3,39_ = 0.31, *P*_ANOVA_ = 0.8; Extended Data Fig. 2) and at the end (*F*_3,38_ = 0.26, *P*_ANOVA_ = 0.9; Extended Data Fig. 2) of the hyperinsulinemic–euglycemic clamp experiments.

There were no interactions between the administered type of nasal spray and cycle phase on insulin courses (*t*_514_ = −0.55, *P*_time × spray × phase_ = 0.6) and on achieved glucose courses (*t*_1,025_ = −1.34, *P*_time × spray × phase_ = 0.2) over the whole hyperinsulinemic–euglycemic clamps. The courses of C-peptide (*t*_514_ = −1.51, *P*_time × spray × phase_ = 0.1) and free fatty acid suppression (*t*_172_ = 0.02, *P*_time × spray × phase_ = 1.0) were also comparable between days.

Regarding our primary research question, spray-induced changes in glucose infusion rates were significantly different between cycle phases (*t*_1,075_ = 2.85, *P*_time × spray × phase_ = 0.004). This remained significant after adjustment for plasma glucose and insulin concentrations (*t*_464_ = 2.53, *P*_time × spray × phase_ = 0.01).

**Fig. 2:**
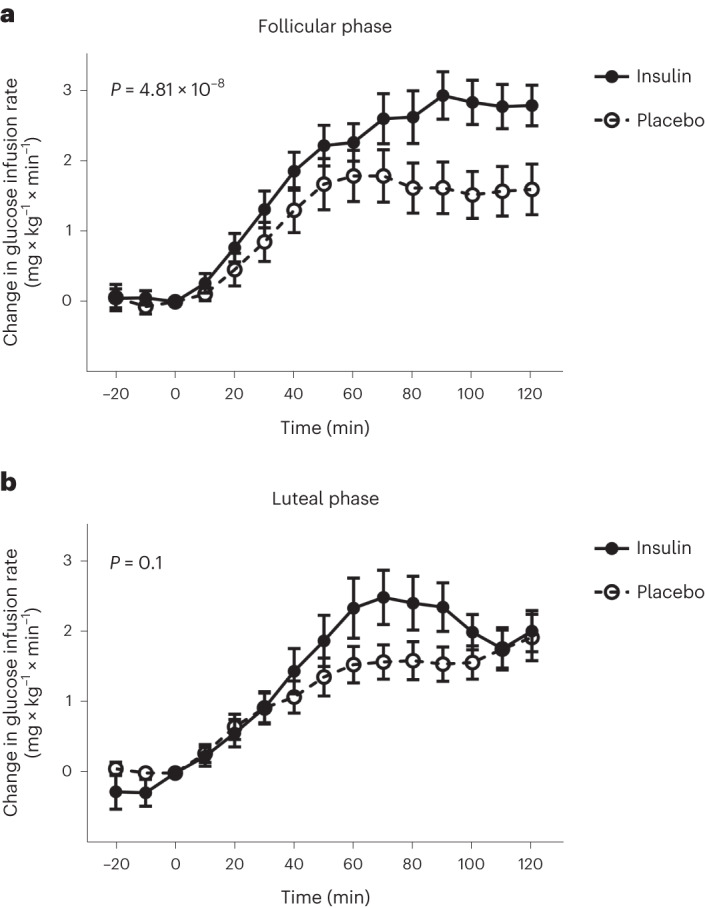
Impact of insulin versus placebo spray on glucose infusion rates in both phases of the menstrual cycle. **a**,**b**, Absolute changes in glucose infusion rates after administration of intranasal insulin (filled circles) or intranasal placebo and i.v. insulin bolus (open circles). Results in the follicular (**a**) and luteal (**b**) phases are shown. *P* values are for time × spray interactions tested by two-sided linear mixed models adjusted for serum insulin and glucose; presented are means, and error bars represent s.e.m.; *N* = 11 participants except for the follicular phase where *N* = 10 for the insulin spray.

In the follicular phase, the glucose infusion rate had to be increased more after intranasal insulin administration than after administration of placebo spray (*t*_525_ = −6.83, *P*_time × spray_ < 0.0001; Fig. 2a). This was independent of insulin and glucose concentrations (*t*_230_ = −5.66, adjusted *P*_time × spray_ < 0.0001).

In the luteal phase, the different courses in glucose infusion rate between insulin and placebo spray (*t*_550_ = −2.83, *P*_time × spray_ = 0.005; Fig. 2b) may result from differences in the achieved plasma glucose and insulin concentrations, as they were no longer detectable after respective adjustments (*t*_234_ = −1.69, adjusted *P*_time × spray_ = 0.09).

To test if the different responses to nasal insulin between cycle phases are secondary to specific hormone levels, we tested for interactions between measured hormone levels and the nasal spray effect on glucose infusion rates. Neither estradiol nor progesterone nor 17-OH progesterone showed such interactions (all *P*_time × spray × hormone_ ≥ 0.2). However, the estradiol:progesterone ratio interacted with spray (placebo versus insulin) and time (*t*_210_ = −2.89, *P*_time × spray × hormone_ = 0.004). This model suggests that a high estradiol:progesterone ratio, as present during the follicular phase, is linked to a stronger effect of intranasal insulin on glucose infusion rates than placebo spray.

Furthermore, comparable interactions were detected for testosterone (*t*_1,075_ = 3.7, *P*_time × spray × hormone_ = 0.0002). However, this was not present for calculated free testosterone (*t*_1,075_ = 0.37, *P*_time × spray × hormone_ = 0.7) and was also not present for other tested androgens (androstenedione (*t*_1,075_ = 1.75, *P*_time × spray × hormone_ = 0.08) and DHEA-sulfate (*t*_1,075_ = −0.64, *P*_time × spray × hormone_ = 0.5)).

## Functional magnetic resonance imaging study

We investigated 15 women for the functional magnetic resonance imaging (fMRI) study (Fig. 1b). Just as in study group 1, body weight and body fat contents were comparable between cycle phases (both *P* ≥ 0.2), and there were differences in sex hormones (Supplementary Table 2). Progesterone, 17-OH-progesterone and estradiol concentrations were higher in the luteal phase. FSH and LH levels did not differ between phases. The studied androgens were also comparable between phases, while calculated free testosterone was higher in the follicular phase. Neither fasting glucose nor insulin nor C-peptide levels differed between phases (all *P* ≥ 0.1).

**Fig. 3:**
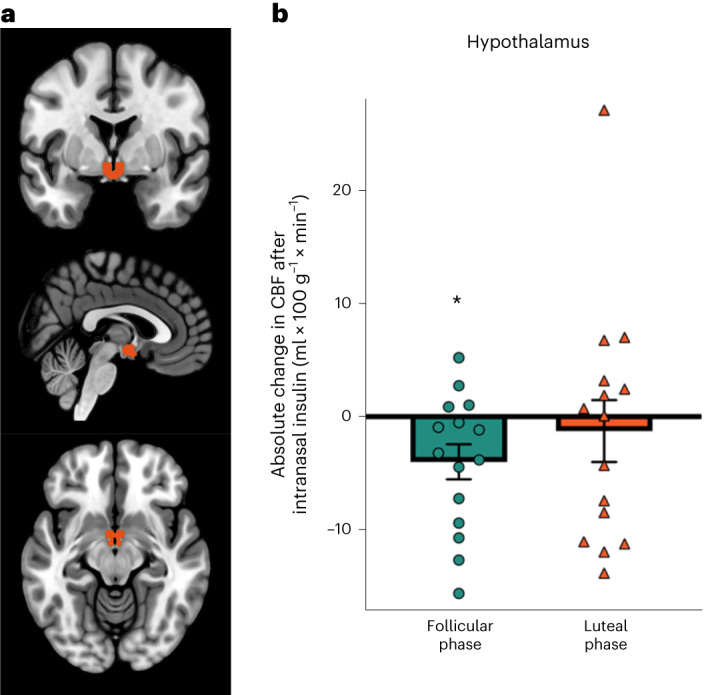
Hypothalamic response to insulin spray in both phases of the menstrual cycle. **a**, Hypothalamus region of interest in coronal (top), sagittal (middle) and axial section planes (bottom). **b**, Absolute changes in CBF in the hypothalamus from before treatment to 30 min after intranasal insulin administration in the follicular (left) and luteal (right) phases of the menstrual cycle. In the follicular phase, insulin induced a significant reduction in hypothalamic CBF, whereas this effect was absent in the luteal phase. Bar graphs are shown with means and individual data points, and error bars represent s.e.m. Data were analysed by two-sided paired *t*-tests; *P = 0.02; *N* = 15 participants in both cycle phases.

On the basis of our previous findings^3,8^, we assessed the insulin-induced response of hypothalamic cerebral blood flow (CBF) as a readout for hypothalamic insulin action. For this purpose, a hypothalamus mask was created based on the WFU pickatlas, which resulted in a mean CBF of 23 voxels. Insulin administration significantly decreased hypothalamic CBF only in the follicular phase from before administration to 30 min after nasal spray application (*t*_14_ = −2.57, *P* = 0.02). In the luteal phase, insulin induced no such effect (*t*_14_ = −0.467, *P* = 0.648; Fig. 3a,b). However, no significant interaction was observed between cycle phase and spray application (*P* = 0.5). Furthermore, no significant correlations with the tested sex hormones were observed for hypothalamic insulin response (*P* ≥ 0.1).

In the follicular phase of the menstrual cycle, brain insulin action improved peripheral insulin sensitivity in lean women, similar to what has previously been described in lean men. Such a response was not detectable in the luteal phase. In our functional neuroimaging study, consistent findings were obtained with modulation of hypothalamic activity by insulin in the follicular phase but not in the luteal phase. However, insulin response in the hypothalamus was not statistically different between cycle phases.

Our findings demonstrate that brain insulin sensitivity is not a fixed trait but can be rapidly regulated, with metabolic consequences for the entire body. The current study corroborates that insulin action in the human brain modulates peripheral insulin sensitivity^3,4,11^ and demonstrates that this mechanism is not limited to lean men but is also present in lean women. This underscores the brain’s function in ‘fine-tuning’ whole-body metabolism to rapidly adapt peripheral energy fluxes to current energy availability^2^.

However, in lean women, this function of insulin in the brain appears to be evident only in the follicular phase. Here, the effects on peripheral insulin sensitivity were comparable in timing and magnitude to previous findings obtained in lean men^3,4^.

How this would relate to women who are overweight/obese is unclear. Most likely, they would behave as men with obesity^3,4,27^ and would not respond to brain insulin delivery, regardless of their menstrual cycle phase. This hypothesis is in line with neuroimaging studies that demonstrated hypothalamic insulin resistance in individuals of both sexes who were overweight or obese^8,28^. However, appropriate interventional studies are needed to experimentally investigate this assumption.

Peripheral insulin sensitivity changes during the menstrual cycle, with relative peripheral insulin resistance occurring in the luteal phase compared to in the follicular phase^29^. Our results suggest that changes in brain insulin responsiveness could be one mechanism that contributes to this phenomenon. The failure of brain insulin to exert its effects on peripheral insulin sensitivity in the luteal phase is likely due to relative hypothalamic insulin resistance in this cycle phase. In line with experimental evidence from animals^30^, previous correlational findings from humans indicate a crucial role of insulin action in the hypothalamus for the modulation of whole-body glucose metabolism^3,4^. Other brain regions may also contribute to this complex regulation^2^. Based on our current results, we hypothesize that hypothalamic insulin resistance in the luteal phase may be responsible for cycle-dependent differences in brain-derived modulation of peripheral insulin sensitivity.

The hypothalamus is crucial not only for peripheral insulin sensitivity and whole-body glucose homeostasis but also for upstream regulation of sex hormones and thereby the menstrual cycle. However, both functions depend on distinct loci of the hypothalamus. The precise anatomical and functional interplay between gonadotropin-releasing hormone-producing neurons in the preoptic area and neurons crucial for whole-body glucose homeostasis in the medial hypothalamus is still largely unexplored. Of note, brain-specific deletion of insulin receptors altered physiologic gonadotropin secretion in rodents of both sexes^31^, suggesting a reciprocal interplay of brain insulin and the menstrual cycle.

It is tempting to speculate on the potential physiologic functions of regulating brain insulin sensitivity across the menstrual cycle. One major function of the menstrual cycle is the preparation of the uterus and the rest of the body for a potential upcoming pregnancy. During the follicular phase, glucose fluxes over the human body must be precisely relayed to areas with high energy need, including the uterus with the growing endometrium. Besides modulating whole-body energy fluxes^2^, insulin sensitivity is crucial for both the uterus and ovaries^32^. Insulin signalling plays a critical role in the proliferation of the endometrium^33^ and development of the preovulatory follicle^34^. Of note, rodents selectively lacking brain insulin receptors display impaired follicular maturation^31^, underpinning the crucial role of brain insulin action in oocyte maturation. Proper brain-derived enhancement of peripheral insulin sensitivity may support insulin-sensitive processes in the uterus and ovaries. These brain insulin-mediated mechanisms are thought to be especially important in the postprandial state^12^ when vast amounts of energy must be guided through the body.

In the luteal phase, lack of the brain-derived modulation of peripheral insulin sensitivity could promote energy storage in adipose tissue, as suggested by findings of progressive fat accumulation in humans with brain insulin resistance^18^. Besides the major effects of sex hormones, these natural changes in brain-derived modulation of adipose energy storage throughout the menstrual cycle could be one contributor to differences in body fat distribution between sexes^35^.

Although we detected an association between testosterone levels and the brain’s ability to enhance peripheral insulin sensitivity, the lack of association with free testosterone argues against a major role in the regulation of brain insulin sensitivity. However, estradiol:progesterone ratio was linked to brain-derived improvement of peripheral insulin sensitivity. This is contrary to findings in healthy men, where estrogen administration did not alter the response in eating behaviour after administration of nasal insulin^36^. However, influences of sex hormones on insulin sensitivity have long been suspected^37^, and sex-specific effects of gonadal hormones are plausible. Precise molecular mechanisms of potential hormonal influences on brain insulin responsiveness need to be further tested in upcoming studies.

We studied only a limited number of participants and did not include women taking oral contraceptives. The detected differences are rather small and come with large confidence intervals, and replication is necessary before final conclusions can be drawn. Furthermore, nasal insulin delivery is not the physiological route. Despite the lack of data on the exact amount of insulin reaching the brain, previous trials found that the used dose of 160 U resulted in substantial effects on brain activity^6,7^, comparable to oral glucose ingestion, and this dose has been widely used in previous trials on acute brain effects of insulin^38^. While we mimicked spillover of nasal insulin into the systemic circulation with statistically comparable circulating concentrations between study days, there was still variation in individual study participants that might potentially have impacted our results. The limited sample size might also underlie the fact that differences in hypothalamic insulin response did not reach statistical significance, while significant interactions between spray and cycle phase were detected in the hyperinsulinemic–euglycemic clamp experiments. In our hyperinsulinemic–euglycemic clamp experiments, we unfortunately did not achieve stable tracer enrichment of d-[6,6-^2^H_2_]-glucose and were therefore not able to disentangle endogenous glucose production and glucose disappearance. Previous findings from healthy men indicate that brain insulin most likely modulates both^3^. Furthermore, our experiments were performed in a single-blind fashion, and the investigators were aware of the condition (nasal insulin versus placebo). In our fMRI experiment, no placebo administration was used as a control condition. However, in our previous placebo-controlled assessments^6,8,39^ (see Supplementary Material 2 for more details), the placebo nasal spray did not impact hypothalamic blood flow.

Taken together, insulin delivery to the brain improves peripheral insulin sensitivity in lean women, comparable to what has previously been described in lean men. However, this response was limited to the follicular phase of the menstrual cycle and appears to be absent in the luteal phase. The underlying mechanism most likely involves hypothalamic insulin resistance during the luteal phase. Brain insulin resistance could therefore contribute to the long-known peripheral insulin resistance in the luteal phase of the menstrual cycle. This could also be involved in worse glycemic control in the luteal phase in women with type I diabetes^40^. Our findings need to be taken into consideration both in mechanistic studies as well as in upcoming therapeutic approaches that address insulin action in the brain.

## Methods

The study (NCT03929419) received approval by the local ethics committee (Ethics Committee of the Medical Faculty of the Eberhard Karls University and the University Hospital of Tübingen) and was conducted according to the relevant guidelines and regulations. Data acquisition was performed at the University Hospital of Tübingen between April 2019 and March 2021.

### Study design and participants

**Extended Data Fig. 3:**
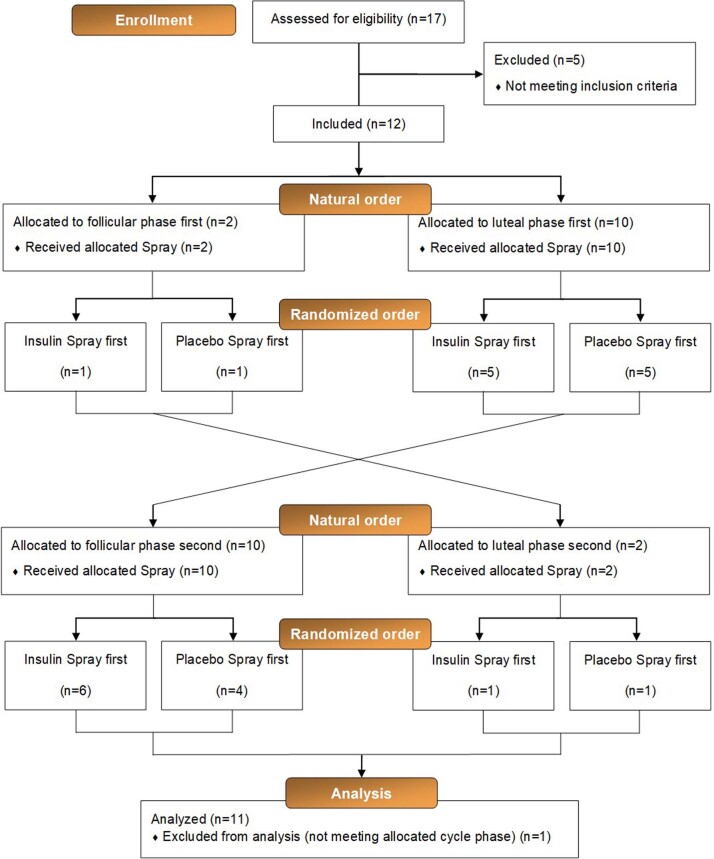
CONSORT flow diagram - hyperinsulinemic-euglycemic clamp study. CONSORT flow chart of the study course of the hyperinsulinemic-euglycemic clamp study.

**Extended Data Fig. 4:**
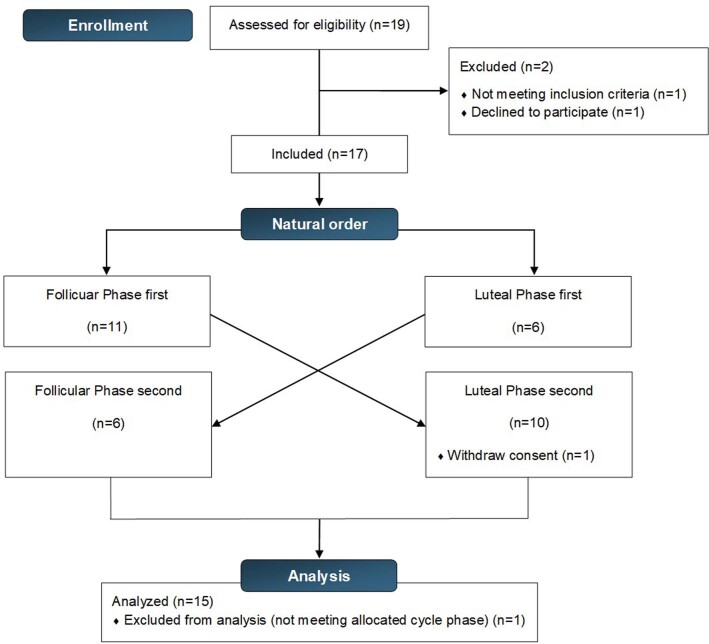
CONSORT flow diagram – fMRI study. CONSORT flow chart of the study course of the fMRI study.

We analysed two different study populations. In the first group (*N* = 11), we performed two clamp experiments per cycle phase using a single-blind crossover design (Extended Data Fig. 3). The second group (*N* = 15) was subjected to one fMRI experiment per cycle phase (Extended Data Fig. 4). The initial cycle phase for the experiments was chosen in a random order. For both groups, young, healthy, naturally cycling women who neither took hormonal contraceptives nor any other medication were recruited (for participant characteristics, see Table 1; for inclusion and exclusion criteria, see Supplementary Table 3). After providing informed written consent, participants were screened for eligibility, where medical history and a blood count were obtained. To ensure a regular menstrual cycle, participants provided information about the length of their last three cycles, which most of them tracked via an app.

**Table 1:** Participant characteristics

Median (IQR)	Hyperinsulinemic–euglycemic clamp study (*N* = 11)	fMRI study (*N* = 15)
Age (years)	24.0 (4.0)	23.0 (4.0)
BMI (kg m^–^²)	21.8 (2.4)	21.8 (2.0)
Body fat content (%)	26.4 (6.5)	29.4 (6.2)^a^
HbA1c (%)	5.2 (0.4)	5.1 (0.3)
HbA1c (mmol mol^–1^)	33.0 (5.0)	33.0 (3.5)
Fasting plasma glucose (mmol liter^–1^)	4.6 (0.77)	4.7 (0.39)
Menstrual cycle duration (d)	29.0 (5.0)	29.0 (2.5)

Data are presented as median (interquartile range (IQR)).

BMI, body mass index; HbA1c, hemoglobin A1c.

^a^Available for *N* = 14 participants.

The respective phase of the menstrual cycle was identified using calendar-based counting and serum sex hormone analysis^41^. Taking the individual cycle length into account, the start of the current and subsequent cycles were recorded to determine the cycle day of the experiment retrospectively. For this, the onset of their last menstruation was considered the first day of the current cycle and denoted the start of the follicular phase. The follicular phase was assumed starting 2 d after the start of the current menstrual cycle but no more than 17 d apart from the start of the subsequent cycle. The luteal phase was assigned in the time frame of a maximum of 10 d but no less than 3 d before the start of the subsequent cycle (Fig. 1). For final cycle phase verification, sex hormones (LH, FSH, estradiol and progesterone) were analysed, as proposed previously^41^.

Participants were instructed to restrain from heavy physical activity, smoking and alcohol within the 24 h before the experiments.

### Hyperinsulinemic–euglycemic clamp (performed in study group 1)

The applied protocol was similar to our previous hyperinsulinemic–euglycemic clamps in men^3,4^. Experiments were conducted in the morning after an overnight fast. Participants underwent two hyperinsulinemic–euglycemic clamp experiments per cycle phase in a single-blind randomized order. Hence, only study participants were blinded for the type of spray, whereas study personnel were not blinded due to safety reasons. A venous cannula was placed in one arm that was warmed to facilitate arterialized blood sampling. Another cannula was placed into the contralateral antecubital vein for infusions. The experiment started with an intravenous insulin bolus of 6.25 mU × kg^−1^, followed by constant i.v. insulin infusion at 0.25 mU × kg^−1^ × min^−1^ for 3.5 h (Insuman Rapid, Sanofi). Nasal spray was administered 1.5 h after initiation of insulin infusion (time point: 0 min). On one day, participants received 160 U of human insulin (eight puffs in each nostril over 4 min; insulin Actrapid, Novo Nordisk), and vehicle was administered as placebo on the other day. Together with placebo spray, insulin infusion was increased by 0.17 mU × kg^−1^ × min^−1^ for 15 min after the first placebo spray puff. This resulted in an i.v. insulin bolus of 2.5 mU × kg^−1^ over 15 min and was performed to mimic spillover of nasal insulin into the systemic circulation, as done previously^3,42^.

During the experiment, blood samples were taken every 5 min to measure blood glucose, and the infusion rate of 20% dextrose was adjusted to maintain euglycemia with a targeted glucose concentration of 5 mmol litr^–1^. Additional blood samples were taken to determine the concentrations of other hormones. The infused glucose was enriched with d-[6,6-^2^H_2_]-glucose, although no stable levels were achieved, and tracer enrichment was therefore not further statistically analysed.

In this paper, the time of spray administration was used as time point 0 min. This makes it easier to understand the timeline of the effects of nasal insulin versus placebo and to facilitate comparison with previous studies using the same clamp protocol in men^3^. As a result, the nomenclature of the time frame in ClinicalTrials.gov and the paper differs, with the time frame 60–90 min after clamp initiation in ClinicalTrials.gov referring to the time before spray administration (that is 0 min in the paper) and the time frame 150–210 min in ClinicalTrials.gov referring to the end of the clamps, which corresponds to 90–120 min in this paper.

#### Analytic procedures

Blood glucose was measured immediately on site by the glucose oxidase method (Biosen C-Line, EKF Diagnostics). Technical errors prevented valid measurement of seven glucose values, which were therefore excluded from analyses. Except for androstenedione, which was measured from samples stored at −80 °C, all other analytes were handled on ice and measured immediately in the central laboratory of the University Hospital of Tübingen. Concentrations of circulating testosterone, insulin, C-peptide, DHEA-sulfate, LH, FSH, cortisol, estradiol and prolactin were measured on an ADVIA Centaur XPT, and sexual hormone-binding globulin and androstenedione were measured on IMMULITE immunoassay systems (Siemens Healthineers). Anti-Müllerian hormone concentrations were determined using the cobas e411 analyser (Roche Diagnostics). Plasma concentrations of albumin as well as total non-esterified fatty acids (enzymatic method, WAKO Chemicals) were measured using the ADVIA XPT clinical chemical analyser (Siemens Healthineers). HbA1c measurements were performed using a Tosoh glycohemoglobin analyser (HLC-723G8, Tosoh Bioscience). 17-OH-Progesterone was measured on the IDS-iSYS Immunoanalyzer (Immunodiagnostic Systems). Based on serum testosterone, sexual hormone-binding globulin and albumin levels, free testosterone in serum was calculated as previously reported^43^.

Hormone measurements were performed in a routine diagnostic laboratory under accreditation with the German accredited body (DAkkS). Internal and external quality control was performed at all times during the study, including proficiency testing four times per year, and passed at all times.

### Brain fMRI measurement (performed in study group 2)

After completing the hyperinsulinemic–euglycemic clamp experiments, we decided to follow up by studying insulin effects in the hypothalamus. However, once the amendment was approved by the Ethics Committee, we were unable to retain all initial participants of the clamp study and therefore recruited 15 new participants who underwent pulsed arterial spin labelling measurement to determine CBF. After the basal fMRI measurement, 160 U of nasal insulin were administered. A second fMRI measurement was performed 30 min later.

#### Data acquisition

Scanning was conducted with a 3T whole-body Siemens scanner (MAGNETOM Prisma) with a 20-channel coil.

As recently reported^8,44^, pulsed arterial spin labelling images were obtained with a PICORE-Q2TIPS (proximal inversion with control for off-resonance effects (quantitative imaging of perfusion by using a single subtraction sequence)) by applying a frequency offset corrected inversion pulse and echo planar imaging readout for acquisition. In addition, high-resolution T1-weighted anatomical images were obtained.

#### Arterial spin labelling image processing

For image preprocessing, ASLtbx with SPM12 (Wellcome Trust Centre for Neuroimaging) was implemented. In detail, functional images were motion corrected, co-registered to the individual anatomical image and smoothed with a full-width at half-maximum of 6 mm. Perfusion images were generated by calculating the control tag differences by using surround subtraction. We used a unique *M*_0_ value extracted from a region of interest in the cerebrospinal fluid to quantify CBF (ml × 100 g^−1^ × min^−1^) and applied the general kinetic model for absolute perfusion quantification. CBF was extracted from the hypothalamus as our region of interest, based on recent findings^3,8^.

### Safety

We observed a short-lasting burning sensation in the nose immediately after administration of both types of nasal sprays in most participants. No other adverse effects were observed. Importantly, there were no cases of allergic reactions or severe hypoglycemia.

### Statistical analysis

Statistical analyses for clinical characteristics and the hyperinsulinemic–euglycemic clamp as well as block randomization for the clamp study were performed using R 4.2.1 (ref. ^45^). Normal distribution was tested by visual inspection of QQ-plots.

Differences in metabolite/hormone levels across study days were tested by one-way analysis of variance (ANOVA). The courses of dynamic variables, such as glucose infusion rates, across clamps were modelled for all time points. We tested interactions between elapsed time during the clamp (standardized) and the appropriate grouping variables (phase: follicular versus luteal; spray: placebo versus insulin) using linear mixed models with the interaction terms as fixed effects. In some models, additional adjustment for plasma glucose and insulin was performed, as indicated. The participants and clamp order of the four investigations were included as random intercept and random slope, respectively. Glucose infusion rates were tested relative to the glucose infusion rate before spray application at 90 min of the clamp. We used the lme4 library^46^ (version 1.1-31) to fit mixed linear models, and *P* values were computed using Satterthwaite’s method as implemented in the lmerTest library^47^ (version 3.1-3). Details on the mixed models are provided in Supplementary Table 4.

Mean hypothalamic CBF before and after nasal insulin application was compared by paired *t*-tests (two tailed) for the follicular and luteal phases separately. The interaction between cycle phase and spray application was tested using a linear mixed model with the interaction term as a fixed effect.

As the fMRI study (study group 2) did not include a placebo spray control, we evaluated hypothalamic CBF response before and 30 min after placebo administration based on previous publications^8,39^. No significant change in hypothalamic blood flow was observed in 110 healthy participants (*P* > 0.05). On a whole-brain level, a significant CBF increase was identified only in the occipital region 30 min after placebo spray application (family-wise error-corrected *P* value of <0.05, corrected for multiple comparisons; see Supplementary Material 1). Furthermore, reproducibility and reliability were assessed for the global and hypothalamic CBF measures in participants with two measurement time points separated by 2 to 6 weeks without a lifestyle or pharmaceutical intervention in between (see Supplementary Material 2).

For the hyperinsulinemic–euglycemic clamp study, a sample size of *N* = 10 was calculated (*α* = 0.05, power (1 – β error probability) = 0.9, G*Power) based on an effect size of Cohen’s *d*_z_ = 1.178, which was achieved in our previous work investigating glucose infusion rate in response to intranasal insulin in lean men^20^. To compensate for potential dropouts, *N* = 2 were additionally recruited.

For the fMRI study, the sample size was chosen based on previous studies using fMRI in combination with intranasal insulin versus placebo showing moderate-to-high within-group (*d*_z_ = 0.7) and high between-group differences (*d*_z_ = 0.9) in hypothalamus insulin action^8,48^.

### Reporting summary

Further information on research design is available in the Nature Portfolio Reporting Summary linked to this article.

### Supplementary information

Supplementary Information Supplementary Tables 1–4 and Materials 1 and 2.

Reporting Summary

Supplementary Data 1 Translated final study protocol of the trial.

Supplementary Data 2 CONSORT checklist.

## Data Availability

The data generated during the current study are shared with researchers upon reasonable request. Requests will be promptly reviewed by the Data Access Steering Committee of the Institute of Diabetes Research and Metabolic Diseases, Tübingen, Germany. Any data and materials that can be shared will be released via a material transfer agreement.
