# Supplementary material for: Brain insulin action on peripheral insulin sensitivity in women depends on menstrual cycle phase
Source: Nat Metab. 2023 Sep 21;5(9):1475–82. doi: 10.1038/s42255-023-00869-w (PMC10513929; doi:10.1038/s42255-023-00869-w)
Supplement: Supplementary file 1 — Supplementary Tables 1–4 and Materials 1 and 2. [file 42255_2023_869_MOESM1_ESM.pdf]

nature metabolism

Letter

https://doi.org/10.1038/s42255-023-00869-w

Brain insulin action on peripheral insulin sensitivity in women depends on menstrual cycle phase

In the format provided by the authors and unedited

Supplementary Table S1: Hormones in the hyperinsulinemic-euglycemic clamp study

N=11	**Follicular phase**	**Luteal phase**	**P-value**
Estradiol (pmol/l)	206 (95.50)	524 (250)	0.07
Progesterone (nmol/l)	1.40 (0.60)	28.4 (34.30)	**0.002**
17-α-OH-progesterone (nmol/l)	1.80 (0.70)	5.50 (2.40)	**0.001**
Follicle-stimulating hormone (IU/l)	6.00 (1.50)	2.70 (1.10)	**0.008**
Luteinizing hormone (IU/l)	4.80 (1.50)	3.50 (3.15)	0.5
Prolactin (µg/l)	13.0 (8.50)	14.0 (5.50)	0.4
Testosterone (nmol/l)	1.00 (0.20)	0.90 (0.20)	0.7
Calculated free testosterone (pmol/l)	11.0 (5.50)	10.0 (3.00)	0.3
Dehydroepiandrosterone-sulfate (µmol/l)	3.50 (1.50)	4.70 (1.90)	**0.01**
Androstenedione (nmol/l)	11.0 (3.00)	13.0 (5.00)	**0.04**
Anti-Müllerian hormone (ng/ml)	2.87 (1.49)	3.20 (2.01)	0.1
Morning cortisol, serum (nmol/l)	462 (98.50)	494 (109)	0.3

Data are presented as Median (IQR). p-values are from two-sided paired t-tests. $p < 0.05$ are printed in bold.

Supplementary Table S2: Hormones in the fMRI study

N=15	**Follicular phase**	**Luteal phase**	**P-value**
Estradiol (pmol/l)	264 (173)	566 (361)	**0.002**
Progesterone (nmol/l)	2.00 (0.70)	28.5 (25.20)	**0.0002**
17-α-OH-progesterone (nmol/l)	5.00 (2.40)	8.80 (3.45)	**0.0002**
Follicle-stimulating hormone (IU/l)	7.40 (1.85)	3.30 (2.25)	0.1
Luteinizing hormone (IU/l)	6.90 (2.40)	7.80 (3.45)	0.3
Prolactin (µg/l)	15.0 (13.50)	16.0 (23.5)	**0.07**
Testosterone (nmol/l)	1.20 (0.55)	1.20 (0.60)	0.2
Calculated free testosterone (pmol/l)	16.0 (8.50)	13.0 (5.50)	**0.01**
Dehydroepiandrosterone-sulfate (µmol/l)	6.70 (3.50)	5.50 (2.70)	0.3
Androstenedione (nmol/l)	15.0 (5.00)	13.0 (4.50)	0.6
Anti-Müllerian hormone (ng/ml)	3.41 (3.62)	2.79 (3.25)	**0.002**
Morning cortisol, serum (nmol/l)	622 (152)	579 (141)	0.3

Data are presented as Median (IQR). p-values are from two-sided paired t-tests. $p < 0.05$ are printed in bold.

Supplementary Table S3: In- and exclusion criteria

**Inclusion criteria**	**Exclusion criteria**
• Female volunteer adults<br>• HbA1c <6.0%<br>• Age between 18 and 30 years<br>• Standard routine laboratory<br>• No underlying diseases<br>• No medication<br>• No hormonal contraception<br>• Understanding of the explanations of the study and the instructions	• Persons carrying non-removable metal parts in or on the body<br>• Persons with limited temperature perception and / or increased temperature sensitivity to warming of the body<br>• Cardiovascular disease, such as manifest coronary heart disease, heart failure greater than NYHA 2, recent myocardial infarction<br>• People with a hearing disorder or increased sensitivity to loud sounds<br>• Persons with claustrophobia<br>• Minors or non-consenting subjects are also excluded<br>• Pregnancy or breastfeeding women<br>• Surgery less than 3 months ago<br>• Simultaneous participation in other intervention studies<br>• Acute illness or infection within the last 4 weeks<br>• Neurological and psychiatric disorders<br>• Subjects with hemoglobin Hb <12g / dl (at screening)<br>• Allergic diseases<br>• Individuals with a history of heparin-induced thrombocytopenia (HIT)

Supplementary Table S4: Results from the hyperinsulinemic-euglycemic clamp study: Summary of two-sided linear mixed models

	Serum insulin concentrations								
Predictors	*Estimates*	*std. Error*	*std. Beta*	*standardized std. Error*	*CI*	*standardized CI*	*p*	*std. p*	*df*
(Intercept)	152.57	4.76	-0.02	0.11	143.05 – 162.09	-0.24 – 0.20	**2.2×10^{-40}**	0.855	62.02
Time*	-9.72	2.58	-0.24	0.06	-14.79 – -4.65	-0.37 – -0.12	**0.0002**	**0.0002**	478.44
Spray [p]	-13.49	5.60	-0.31	0.13	-24.62 – -2.36	-0.56 – -0.06	**0.018**	**0.017**	84.35
Phase [2]	10.43	7.88	0.24	0.18	-5.67 – 26.54	-0.13 – 0.60	0.196	0.197	29.64
Time* × Spray [p]	1.82	3.57	0.05	0.09	-5.19 – 8.82	-0.13 – 0.22	0.610	0.610	478.44
Time* × Phase [2]	0.56	3.57	0.01	0.09	-6.44 – 7.57	-0.16 – 0.19	0.875	0.875	478.44
Spray [p] × Phase [2]	-9.45	8.99	-0.21	0.20	-27.61 – 8.71	-0.62 – 0.20	0.299	0.310	39.80
(Time* × Spray [p]) × Phase [2]	-2.73	4.98	-0.07	0.13	-12.52 – 7.07	-0.31 – 0.18	0.585	0.585	478.46
**Random Effects**									
σ^2	980.65								
$\tau_{00\ id}$	176.15								
$\tau_{11\ id.order2}$	433.30								
$\tau_{11\ id.order3}$	2938.83								
$\tau_{11\ id.order4}$	99.50								
ρ_{01}	-0.66								
	-0.68								
	-0.71								
ICC	0.15								
N_{id}	11								
Observations	514								
Marginal R^2 / Conditional R^2	0.150 / 0.279								
AIC	5066.175								

* centered (scaled)
p: placebo
2: Follicular phase of the menstrual cycle (2nd phase)

	Plasma glucose concentrations								
Predictors	*Estimates*	*std. Error*	*std. Beta*	*standardized std. Error*	*CI*	*standardized CI*	*p*	*std. p*	*df*
(Intercept)	87.60	0.71	0.06	0.10	86.16 – 89.04	-0.15 – 0.27	**2.5×10^{-51}**	0.558	38.19
Time*	1.47	0.42	0.20	0.06	0.64 – 2.29	0.09 – 0.32	**0.001**	**0.001**	989.17
Spray [p]	0.02	0.69	0.00	0.10	-1.34 – 1.39	-0.19 – 0.20	0.973	0.975	125.98
Phase [2]	0.26	0.78	0.04	0.11	-1.36 – 1.88	-0.19 – 0.27	0.743	0.719	21.91
Time* × Spray [p]	-0.04	0.58	-0.01	0.08	-1.19 – 1.11	-0.17 – 0.15	0.945	0.945	989.28
Time* × Phase [2]	0.76	0.60	0.11	0.08	-0.42 – 1.94	-0.06 – 0.27	0.207	0.207	1001.80
Spray [p] × Phase [2]	-1.24	1.02	-0.18	0.15	-3.31 – 0.83	-0.48 – 0.11	0.233	0.219	33.78
(Time* × Spray [p]) × Phase [2]	-1.12	0.83	-0.16	0.12	-2.76 – 0.52	-0.38 – 0.07	0.180	0.180	999.32
**Random Effects**									
σ^2	41.87								
$\tau_{00\ id}$	2.84								
$\tau_{11\ id.order2}$	5.85								
$\tau_{11\ id.order3}$	1.74								
$\tau_{11\ id.order4}$	3.54								
ρ_{01}	-0.16								
	-0.17								
	-0.58								
N_{id}	11								
Observations	1025								
Marginal R^2 / Conditional R^2	0.059 / NA								
AIC	6810.201								

* centered (scaled)

	Serum C-peptide concentrations								
Predictors	*Estimates*	*std. Error*	*std. Beta*	*standardized std. Error*	*CI*	*standardized CI*	*p*	*std. p*	*df*
(Intercept)	241.41	13.13	-0.60	0.10	210.57 – 272.25	-0.83 – -0.36	**$2.4x 10^{-7}$**	**0.001**	7.22
Time*	-1.14	4.69	-0.01	0.04	-10.36 – 8.09	-0.09 – 0.07	0.808	0.808	471.00
Spray [p]	89.93	14.89	0.70	0.12	55.36 – 124.49	0.43 – 0.97	**0.0004**	**0.0004**	7.67
Phase [2]	98.84	29.03	0.76	0.23	38.59 – 159.10	0.29 – 1.23	**0.003**	**0.003**	21.67
Time* × Spray [p]	-1.66	6.49	-0.01	0.06	-14.40 – 11.09	-0.12 – 0.10	0.798	0.798	471.00
Time* × Phase [2]	15.55	6.49	0.13	0.06	2.81 – 28.30	0.02 – 0.24	**0.017**	**0.017**	471.00
Spray [p] × Phase [2]	-94.00	33.16	-0.72	0.26	-162.59 – -25.41	-1.25 – -0.19	**0.009**	**0.010**	23.10
(Time* × Spray [p]) × Phase [2]	-13.65	9.07	-0.12	0.08	-31.46 – 4.17	-0.27 – 0.04	0.133	0.133	471.02
**Random Effects**									
σ^2	3246.03								
$\tau_{00\ id}$	15322.72								
$\tau_{11\ id.order2}$	12462.40								
$\tau_{11\ id.order3}$	32538.54								
$\tau_{11\ id.order4}$	17422.48								
ρ_{01}	-0.96								
	-0.38								
	-0.84								
ICC	0.83								
N_{id}	11								
Observations	514								
Marginal R^2 / Conditional R^2	0.082 / 0.840								
AIC	5737.508								

* centered (scaled)

	Circulating non-esterified fatty acids								
Predictors	*Estimates*	*std. Error*	*std. Beta*	*standardized std. Error*	*CI*	*standardized CI*	*p*	*std. p*	*df*
(Intercept)	65.75	9.12	-0.09	0.18	46.91 – 84.60	-0.47 – 0.30	**$2.3x 10^{-7}$**	0.646	19.64
Time*	-8.23	4.05	-0.22	0.11	-16.24 – -0.21	-0.44 – -0.01	**0.044**	**0.044**	137.60
Spray [p]	-12.03	10.83	-0.09	0.21	-33.52 – 9.46	-0.51 – 0.33	0.269	0.678	85.71
Phase [2]	13.96	10.79	0.24	0.21	-7.47 – 35.39	-0.18 – 0.66	0.199	0.256	78.89
Time* × Spray [p]	11.69	5.60	0.32	0.15	0.62 – 22.76	0.02 – 0.62	**0.039**	**0.039**	137.60
Time* × Phase [2]	-3.59	5.60	-0.10	0.15	-14.66 – 7.48	-0.40 – 0.20	0.523	0.523	137.60
Spray [p] × Phase [2]	-3.69	16.20	-0.07	0.32	-35.74 – 28.35	-0.71 – 0.56	0.820	0.816	120.19
(Time* × Spray [p]) × Phase [2]	0.19	7.82	0.01	0.21	-15.28 – 15.66	-0.41 – 0.42	0.980	0.980	137.60
**Random Effects**									
σ^2	1093.13								
$\tau_{00\ id}$	1730.14								
$\tau_{11\ id.order2}$	1214.17								
$\tau_{11\ id.order3}$	3117.29								
$\tau_{11\ id.order4}$	2876.38								
ρ_{01}	-0.89								
	-0.87								
	-0.65								
ICC	0.61								
N_{id}	11								
Observations	172								
Marginal R^2 / Conditional R^2	0.043 / 0.629								
AIC	1745.231								

* centered (scaled)

	Change in glucose infusion rate after spray administration								
Predictors	*Estimates*	*std. Error*	*std. Beta*	*standardized std. Error*	*CI*	*standardized CI*	*p*	*std. p*	*df*
(Intercept)	1.60	0.21	0.13	0.18	1.16 – 2.04	-0.24 – 0.50	**$1.9x 10^{-7}$**	0.471	21.58
Time*	0.90	0.04	0.73	0.03	0.82 – 0.99	0.67 – 0.80	**$9.0x 10^{-84}$**	**$9.0x 10^{-84}$**	1032.00
Spray [p]	-0.57	0.18	-0.50	0.15	-0.96 – -0.18	-0.82 – -0.17	**0.007**	**0.005**	14.44
Phase [2]	-0.25	0.17	-0.22	0.15	-0.62 – 0.11	-0.53 – 0.08	0.166	0.145	17.86
Time* × Spray [p]	-0.40	0.06	-0.32	0.05	-0.51 – -0.28	-0.41 – -0.23	**$2.2x 10^{-11}$**	**$2.2x 10^{-11}$**	1032.00
Time* × Phase [2]	-0.22	0.06	-0.18	0.05	-0.34 – -0.11	-0.27 – -0.09	**0.0002**	**0.0002**	1032.00
Spray [p] × Phase [2]	0.31	0.26	0.27	0.22	-0.23 – 0.84	-0.18 – 0.71	0.248	0.226	24.87
(Time* × Spray [p]) × Phase [2]	0.23	0.08	0.19	0.07	0.07 – 0.39	0.06 – 0.32	**0.004**	**0.004**	1032.00
**Random Effects**									
σ^2	0.42								
$\tau_{00\ id}$	0.29								
$\tau_{11\ id.order2}$	0.59								
$\tau_{11\ id.order3}$	0.34								
$\tau_{11\ id.order4}$	0.46								
ρ_{01}	-0.10								
	-0.06								
	-0.28								
ICC	0.41								
N_{id}	11								
Observations	1075								
Marginal R^2 / Conditional R^2	0.397 / 0.644								
AIC	2309.054								

* centered (scaled)

	Change in glucose infusion rate after spray administration with adjustments for glucose and insulin								
Predictors	*Estimates*	*std. Error*	*std. Beta*	*standardized std. Error*	*CI*	*standardized CI*	*p*	*std. p*	*df*
(Intercept)	2.99	0.46	0.17	0.16	2.09 – 3.89	-0.16 – 0.49	**3.0x 10^{-10}**	0.302	18.50
Glucose	-0.02	0.00	-0.13	0.03	-0.03 – -0.01	-0.19 – -0.07	**2.5x 10^{-5}**	**2.5x 10^{-5}**	430.20
Insulin	0.00	0.00	0.08	0.04	0.00 – 0.00	0.01 – 0.16	**0.021**	**0.021**	106.94
Time*	0.98	0.06	0.89	0.05	0.86 – 1.10	0.79 – 1.00	**9.9x 10^{-48}**	**9.9x 10^{-48}**	433.40
Spray [p]	-0.37	0.17	-0.33	0.15	-0.73 – -0.02	-0.64 – -0.03	**0.041**	**0.033**	21.06
Phase [2]	-0.27	0.15	-0.24	0.13	-0.58 – 0.04	-0.50 – 0.03	0.086	0.074	19.54
Time* × Spray [p]	-0.41	0.08	-0.38	0.07	-0.57 – -0.26	-0.52 – -0.24	**1.6x 10^{-7}**	**1.6x 10^{-7}**	420.95
Time* × Phase [2]	-0.28	0.08	-0.25	0.07	-0.43 – -0.12	-0.39 – -0.11	**0.0005**	**0.0005**	426.00
Spray [p] × Phase [2]	0.26	0.22	0.23	0.19	-0.19 – 0.71	-0.15 – 0.62	0.249	0.227	26.77
(Time* × Spray [p]) × Phase [2]	0.28	0.11	0.25	0.10	0.06 – 0.49	0.06 – 0.45	**0.012**	**0.012**	423.93
**Random Effects**									
σ^2	0.39								
$\tau_{00\ id}$	0.19								
$\tau_{11\ id.order2}$	0.46								
$\tau_{11\ id.order3}$	0.11								
$\tau_{11\ id.order4}$	0.27								
ρ_{01}	0.04								
	0.22								
	-0.34								
ICC	0.33								
N_{id}	11								
Observations	464								
Marginal R^2 / Conditional R^2	0.475 / 0.650								
AIC	1047.721								

* centered (scaled)

	Change in glucose infusion rate (follicular phase (1))								
Predictors	*Estimates*	*std. Error*	*std. Beta*	*standardized std. Error*	*CI*	*standardized CI*	*p*	*std. p*	*df*
(Intercept)	1.48	0.09	-0.08	0.08	1.29 – 1.67	-0.23 – 0.08	**1.1×10^{-19}**	0.327	41.13
Time*	0.90	0.04	0.70	0.03	0.82 – 0.99	0.64 – 0.77	**2.1×10^{-73}**	**2.1×10^{-73}**	507.23
Spray [p]	-0.41	0.16	-0.35	0.12	-0.72 – -0.10	-0.60 – -0.10	**0.011**	**0.007**	60.59
Time* × Spray [p]	-0.40	0.06	-0.31	0.05	-0.51 – -0.28	-0.40 – -0.22	**2.5×10^{-11}**	**2.5×10^{-11}**	507.23
**Random Effects**									
σ^2	0.41								
$\tau_{00\ id}$	0.00								
$\tau_{11\ id.order2}$	1.08								
$\tau_{11\ id.order3}$	0.48								
$\tau_{11\ id.order4}$	0.69								
ρ_{01}									
N_{id}	11								
Observations	525								
Marginal R^2 / Conditional R^2	0.566 / NA								
AIC	1133.687								

* centered (scaled)

	Change in glucose infusion rate (follicular phase (1)) with adjustments for glucose and insulin								
Predictors	*Estimates*	*std. Error*	*std. Beta*	*standardized std. Error*	*CI*	*standardized CI*	*p*	*std. p*	*df*
(Intercept)	3.48	0.52	0.28	0.10	2.45 – 4.51	0.08 – 0.48	**2.4×10^{-10}**	**0.007**	58.46
Glucose	-0.02	0.01	-0.14	0.04	-0.04 – -0.01	-0.21 – -0.07	**0.0002**	**0.0002**	216.76
Insulin	0.00	0.00	0.09	0.05	-0.00 – 0.00	-0.00 – 0.19	0.063	0.062	200.81
Time*	0.99	0.06	0.84	0.05	0.87 – 1.10	0.74 – 0.94	**7.9×10^{-41}**	**7.9×10^{-41}**	216.66
Spray [p]	-0.62	0.16	-0.51	0.12	-0.94 – -0.31	-0.77 – -0.26	**0.0003**	**0.0002**	34.12
Time* × Spray [p]	-0.42	0.07	-0.35	0.06	-0.56 – -0.27	-0.47 – -0.23	**4.8×10^{-8}**	**4.8×10^{-8}**	211.68
**Random Effects**									
σ^2	0.34								
$\tau_{00\ id}$	0.03								
$\tau_{11\ id.order2}$	0.95								
$\tau_{11\ id.order3}$	1.03								
$\tau_{11\ id.order4}$	0.52								
ρ_{01}	-1.00								
	-1.00								
	-0.65								
N_{id}	11								
Observations	230								
Marginal R^2 / Conditional R^2	0.684 / NA								
AIC	524.444								

* centered (scaled)

	Change in glucose infusion rate (luteal phase(2))								
Predictors	*Estimates*	*std. Error*	*std. Beta*	*standardized std. Error*	*CI*	*standardized CI*	*p*	*std. p*	*df*
(Intercept)	1.62	0.11	0.26	0.09	1.39 – 1.85	0.05 – 0.46	**7.8x 10^{-10}**	**0.017**	13.44
Time*	0.68	0.04	0.58	0.03	0.60 – 0.76	0.52 – 0.65	**8.7x 10^{-51}**	**8.7x 10^{-51}**	530.26
Spray [p]	-0.43	0.14	-0.39	0.13	-0.74 – -0.11	-0.67 – -0.11	**0.012**	**0.010**	10.99
Time* × Spray [p]	-0.16	0.06	-0.14	0.05	-0.28 – -0.05	-0.24 – -0.04	**0.005**	**0.005**	530.26
**Random Effects**									
σ^2	0.43								
$\tau_{00\ id}$	0.40								
$\tau_{11\ id.order2}$	0.10								
$\tau_{11\ id.order3}$	0.32								
$\tau_{11\ id.order4}$	1.12								
ρ_{01}	-0.19								
	-0.13								
	-1.00								
N_{id}	11								
Observations	550								
Marginal R^2 / Conditional R^2	0.479 / NA								

*centered (scaled)

	**Change in glucose infusion rate (luteal phase(2)) with adjustments for glucose and insulin**								
Predictors	*Estimates*	*std. Error*	*std. Beta*	*standardized std. Error*	*CI*	*standardized CI*	*p*	*std. p*	*df*
(Intercept)	2.72	0.69	0.24	0.11	1.37 – 4.08	0.01 – 0.47	**0.0001**	**0.045**	13.43
Glucose	-0.02	0.01	-0.12	0.05	-0.03 – -0.00	-0.21 – -0.02	**0.016**	**0.016**	204.89
Insulin	0.00	0.00	0.08	0.06	-0.00 – 0.00	-0.04 – 0.20	0.194	0.194	64.42
Time*	0.69	0.06	0.70	0.06	0.57 – 0.82	0.57 – 0.83	**5.4×10^{-22}**	**5.4×10^{-22}**	223.00
Spray [p]	-0.35	0.13	-0.33	0.12	-0.62 – -0.07	-0.59 – -0.07	**0.015**	**0.014**	22.73
Time* × Spray [p]	-0.14	0.08	-0.14	0.08	-0.29 – 0.02	-0.30 – 0.02	0.093	0.093	218.07
**Random Effects**									
σ^2	0.42								
$\tau_{00\ id}$	0.28								
$\tau_{11\ id.order2}$	0.01								
$\tau_{11\ id.order3}$	0.02								
$\tau_{11\ id.order4}$	0.55								
ρ_{01}	-0.09								
	0.00								
	-1.00								
N_{id}	11								
Observations	234								
Marginal R^2 / Conditional R^2	0.491 / NA								
AIC	560.250								

* centered (scaled)

	Change in glucose infusion rate, interaction model with estradiol								
Predictors	*Estimates*	*std. Error*	*std. Beta*	*standardized std. Error*	*CI*	*standardized CI*	*p*	*std. p*	*df*
(Intercept)	1.04	0.18	0.08	0.10	0.66 – 1.41	-0.14 – 0.30	**2.3×10^{-6}**	0.451	13.44
Estradiol	0.00	0.00	0.13	0.08	-0.00 – 0.00	-0.03 – 0.29	0.097	0.098	23.75
Time*	0.73	0.08	0.85	0.05	0.57 – 0.90	0.75 – 0.95	**2.8×10^{-15}**	**1.8×10^{-37}**	179.04
Spray [p]	0.10	0.22	-0.24	0.09	-0.34 – 0.54	-0.41 – -0.07	0.663	**0.007**	92.24
Estradiol × Time*	0.00	0.00	0.02	0.05	-0.00 – 0.00	-0.08 – 0.12	0.754	0.754	179.79
Estradiol × Spray [p]	-0.00	0.00	-0.22	0.10	-0.00 – -0.00	-0.44 – -0.01	**0.035**	**0.039**	38.66
Time* × Spray [p]	-0.15	0.12	-0.28	0.07	-0.39 – 0.08	-0.43 – -0.14	0.188	**0.0001**	179.68
(Estradiol × Time*) × Spray [p]	-0.00	0.00	-0.07	0.07	-0.00 – 0.00	-0.22 – 0.08	0.340	0.340	179.69
**Random Effects**									
σ^2	0.36								
$\tau_{00\ id}$	0.16								
$\tau_{11\ id.order2}$	0.16								
$\tau_{11\ id.order3}$	0.08								
$\tau_{11\ id.order4}$	0.12								
ρ_{01}	0.36								
	-0.50								
	-0.52								
ICC	0.31								
N_{id}	11								
Observations	213								
Marginal R^2 / Conditional R^2	0.579 / 0.710								
AIC	540.174								

*centered (scaled)

	Change in glucose infusion rate, interaction model with progesterone								
Predictors	*Estimates*	*std. Error*	*std. Beta*	*standardized std. Error*	*CI*	*standardized CI*	*p*	*std. p*	*df*
(Intercept)	1.35	0.15	0.09	0.12	1.04 – 1.67	-0.16 – 0.34	**7.7x 10^{-9}**	0.435	15.57
Progesterone	-0.00	0.00	-0.07	0.06	-0.01 – 0.00	-0.19 – 0.05	0.200	0.248	26.73
Time*	0.83	0.06	0.86	0.05	0.71 – 0.94	0.76 – 0.97	**2.7x 10^{-30}**	**4.4x 10^{-37}**	171.28
Spray [p]	-0.26	0.13	-0.26	0.09	-0.54 – 0.02	-0.44 – -0.08	0.069	**0.007**	22.24
Progesterone × Time*	-0.00	0.00	-0.10	0.05	-0.01 – -0.00	-0.19 – -0.01	**0.035**	**0.035**	172.10
Progesterone × Spray [p]	-0.01	0.01	-0.09	0.10	-0.02 – 0.01	-0.29 – 0.12	0.417	0.391	19.07
Time* × Spray [p]	-0.29	0.08	-0.31	0.08	-0.45 – -0.13	-0.45 – -0.16	**0.0004**	**7.0x 10^{-5}**	174.52
(Progesterone × Time*) × Spray [p]	0.00	0.00	0.04	0.09	-0.01 – 0.01	-0.14 – 0.21	0.688	0.688	176.88
**Random Effects**									
σ^2	0.36								
$\tau_{00\ id}$	0.15								
$\tau_{11\ id.order2}$	0.13								
$\tau_{11\ id.order3}$	0.05								
$\tau_{11\ id.order4}$	0.04								
ρ_{01}	0.46								
	0.11								
	-0.61								
ICC	0.30								
N_{id}	11								
Observations	213								
Marginal R^2 / Conditional R^2	0.583 / 0.708								
AIC	516.903								

*centered (scaled)

	Change in glucose infusion rate, interaction model with 17-OH-progesterone								
Predictors	*Estimates*	*std. Error*	*std. Beta*	*standardized std. Error*	*CI*	*standardized CI*	*p*	*std. p*	*df*
(Intercept)	1.56	0.23	0.06	0.16	1.08 – 2.04	-0.28 – 0.40	**$6.2x\ 10^{-7}$**	0.713	13.69
17-OH-progesterone	-0.01	0.03	-0.03	0.07	-0.07 – 0.05	-0.17 – 0.11	0.716	0.670	12.97
Time*	0.91	0.05	0.65	0.02	0.82 – 1.01	0.60 – 0.70	**$8.7x\ 10^{-65}$**	**$1.2x\ 10^{-121}$**	1032.00
Spray [p]	-0.26	0.24	-0.38	0.11	-0.76 – 0.23	-0.60 – -0.15	0.281	**0.002**	19.75
17-OH-progesterone × Time*	-0.03	0.01	-0.06	0.02	-0.04 – -0.01	-0.10 – -0.02	**0.002**	**0.002**	1032.00
17-OH-progesterone × Spray [p]	-0.04	0.05	-0.10	0.12	-0.15 – 0.06	-0.35 – 0.15	0.415	0.431	23.55
Time* × Spray [p]	-0.38	0.07	-0.24	0.03	-0.53 – -0.24	-0.30 – -0.17	**$2.1x\ 10^{-7}$**	**$5.1x\ 10^{-12}$**	1032.00
(17-OH-progesterone × Time*) × Spray [p]	0.02	0.02	0.05	0.04	-0.01 – 0.05	-0.02 – 0.12	0.158	0.158	1032.00
**Random Effects**									
σ^2	0.42								
$\tau_{00\ id}$	0.34								
$\tau_{11\ id.order2}$	0.54								
$\tau_{11\ id.order3}$	0.28								
$\tau_{11\ id.order4}$	0.41								
ρ_{01}	-0.05								
	-0.09								
	-0.37								
ICC	0.44								
N_{id}	11								
Observations	1075								
Marginal R^2 / Conditional R^2	0.378 / 0.655								
AIC	2327.176								

*centered (scaled)

	Change in glucose infusion rate, interaction model with estradiol/progesterone ratio								
Predictors	*Estimates*	*std. Error*	*std. Beta*	*standardized std. Error*	*CI*	*standardized CI*	*p*	*std. p*	*df*
(Intercept)	1.25	0.14	0.11	0.11	0.96 – 1.54	-0.14 – 0.35	**$8.1x\ 10^{-8}$**	0.368	15.90
Estradiol/ progesterone ratio	0.00	0.00	0.04	0.11	-0.00 – 0.00	-0.18 – 0.25	0.585	0.726	31.52
Time*	0.64	0.06	0.86	0.05	0.53 – 0.76	0.75 – 0.96	**$1.2x\ 10^{-21}$**	**$8x\ 10^{-38}$**	181.49
Spray [p]	-0.23	0.14	-0.25	0.09	-0.51 – 0.05	-0.42 – -0.08	0.102	**0.005**	83.73
Estradiol/ progesterone ratio × Time*	0.00	0.00	0.18	0.06	0.00 – 0.00	0.05 – 0.31	**0.005**	**0.005**	189.26
Estradiol/ progesterone ratio × Spray [p]	-0.00	0.00	-0.08	0.12	-0.00 – 0.00	-0.33 – 0.17	0.398	0.526	39.15
Time* × Spray [p]	-0.11	0.08	-0.29	0.07	-0.27 – 0.05	-0.43 – -0.14	0.175	**0.0001**	179.51
(Estradiol/ progesterone ratio × Time*) × Spray [p]	-0.00	0.00	-0.23	0.08	-0.00 – -0.00	-0.38 – -0.07	**0.004**	**0.004**	189.98
**Random Effects**									
σ^2	0.35								
$\tau_{00\ id}$	0.18								
$\tau_{11\ id.order2}$	0.19								
$\tau_{11\ id.order3}$	0.07								
$\tau_{11\ id.order4}$	0.11								
ρ_{01}	0.14								
	-0.45								
	-0.59								
N_{id}	11								
Observations	210								
Marginal R^2 / Conditional R^2	0.671 / NA								
AIC	525.752								

*centered (scaled)

	Change in glucose infusion rate, interaction model with testosterone								
Predictors	*Estimates*	*std. Error*	*std. Beta*	*standardized std. Error*	*CI*	*standardized CI*	*p*	*std. p*	*df*
(Intercept)	3.17	0.43	0.21	0.14	2.23 – 4.12	-0.12 – 0.53	**$9.5x\ 10^{-6}$**	0.183	7.53
Testosterone	-1.57	0.41	-0.26	0.07	-2.48 – -0.66	-0.40 – -0.11	**0.003**	**0.003**	10.98
Time*	1.06	0.13	0.64	0.02	0.79 – 1.32	0.59 – 0.69	**$8.5x\ 10^{-15}$**	**$1.7x\ 10^{-121}$**	1032.00
Spray [p]	-2.20	0.55	-0.51	0.08	-3.38 – -1.03	-0.68 – -0.34	**0.001**	**$7.6x\ 10^{-6}$**	18.08
Testosterone × Time*	-0.28	0.14	-0.04	0.02	-0.55 – -0.01	-0.09 – -0.00	**0.040**	**0.040**	1032.00
Testosterone × Spray [p]	1.71	0.60	0.28	0.10	0.44 – 2.98	0.08 – 0.49	**0.011**	**0.010**	15.56
Time* × Spray [p]	-1.04	0.21	-0.22	0.03	-1.45 – -0.63	-0.29 – -0.16	**$7.8x\ 10^{-7}$**	**$4.6x\ 10^{-11}$**	1032.00
(Testosterone × Time*) × Spray [p]	0.81	0.22	0.13	0.03	0.39 – 1.24	0.06 – 0.19	**0.0002**	**0.0002**	1032.00
**Random Effects**									
σ^2	0.42								
$\tau_{00\ id}$	0.56								
$\tau_{11\ id.order2}$	0.72								
$\tau_{11\ id.order3}$	0.29								
$\tau_{11\ id.order4}$	0.62								
ρ_{01}	-0.47								
	-0.66								
	-0.46								
ICC	0.57								
N_{id}	11								
Observations	1075								
Marginal R^2 / Conditional R^2	0.364 / 0.726								
AIC	2296.669								

*centered (scaled)

	Change in glucose infusion rate, interaction model with calculated free testosterone								
Predictors	*Estimates*	*std. Error*	*std. Beta*	*standardized std. Error*	*CI*	*standardized CI*	*p*	*std. p*	*df*
(Intercept)	2.10	0.44	0.11	0.15	1.19 – 3.00	-0.22 – 0.44	**$6.1x 10^{-5}$**	0.495	13.86
Calc. free testosterone	-0.05	0.04	-0.13	0.10	-0.13 – 0.03	-0.34 – 0.07	0.208	0.198	24.89
Time*	0.98	0.10	0.64	0.02	0.79 – 1.16	0.59 – 0.68	**$1.4x 10^{-23}$**	**$6x 10^{-120}$**	1032.00
Spray [p]	-0.78	0.39	-0.38	0.10	-1.63 – 0.06	-0.58 – -0.18	0.067	**0.001**	18.12
Calc. free testosterone × Time*	-0.02	0.01	-0.05	0.02	-0.04 – -0.00	-0.09 – -0.00	**0.037**	**0.037**	1032.00
Calc. free testosterone × Spray [p]	0.03	0.04	0.09	0.10	-0.05 – 0.11	-0.13 – 0.30	0.400	0.396	15.40
Time* × Spray [p]	-0.32	0.15	-0.22	0.03	-0.61 – -0.04	-0.29 – -0.15	**0.026**	**$6.1x 10^{-11}$**	1032.00
(Calc. free testosterone × Time*) × Spray [p]	0.00	0.01	0.01	0.03	-0.02 – 0.03	-0.05 – 0.08	0.714	0.714	1032.00
**Random Effects**									
σ^2	0.42								
$\tau_{00\ id}$	0.39								
$\tau_{11\ id.order2}$	0.58								
$\tau_{11\ id.order3}$	0.27								
$\tau_{11\ id.order4}$	0.53								
ρ_{01}	-0.23								
	-0.36								
	-0.50								
ICC	0.48								
N_{id}	11								
Observations	1075								
Marginal R^2 / Conditional R^2	0.370 / 0.673								
AIC	2331.285								

*centered (scaled)

	Change in glucose infusion rate, interaction model with androstenedione								
Predictors	*Estimates*	*std. Error*	*std. Beta*	*standardized std. Error*	*CI*	*standardized CI*	*p*	*std. p*	*df*
(Intercept)	2.91	0.31	0.12	0.14	2.23 – 3.59	-0.19 – 0.44	**5.8x 10^{-7}**	0.394	9.83
Androstenedione	-0.10	0.02	-0.47	0.10	-0.14 – -0.06	-0.67 – -0.26	**0.0002**	**0.0001**	17.58
Time*	0.84	0.07	0.64	0.02	0.70 – 0.98	0.59 – 0.69	**9x 10^{-31}**	**3.5x 10^{-120}**	1032.00
Spray [p]	-1.56	0.24	-0.49	0.07	-2.07 – -1.05	-0.65 – -0.33	**1.7x 10^{-5}**	**1.1x 10^{-5}**	13.99
Androstenedione × Time*	-0.00	0.00	-0.02	0.02	-0.01 – 0.01	-0.06 – 0.02	0.417	0.417	1032.00
Androstenedione 0 × Spray [p]	0.08	0.02	0.36	0.08	0.04 – 0.11	0.18 – 0.53	**0.001**	**0.001**	12.98
Time* × Spray [p]	-0.45	0.11	-0.22	0.03	-0.66 – -0.24	-0.29 – -0.16	**3.3x 10^{-5}**	**5.4x 10^{-11}**	1032.00
(Androstendion 0 × Time*) × Spray [p]	0.01	0.01	0.06	0.03	-0.00 – 0.03	-0.01 – 0.13	0.081	0.081	1032.00
**Random Effects**									
σ^2	0.43								
$\tau_{00\ id}$	0.49								
$\tau_{11\ id.order2}$	0.88								
$\tau_{11\ id.order3}$	0.44								
$\tau_{11\ id.order4}$	0.57								
ρ_{01}	-0.18								
	-0.46								
	-0.43								
ICC	0.54								
N $_{id}$	11								
Observations	1075								
Marginal R^2 / Conditional R^2	0.427 / 0.734								
AIC	2332.509								

*centered (scaled)

	Change in glucose infusion rate, interaction model with DHEA-sulfate								
Predictors	*Estimates*	*std. Error*	*std. Beta*	*standardized std. Error*	*CI*	*standardized CI*	*p*	*std. p*	*df*
(Intercept)	2.48	0.33	0.04	0.11	1.72 – 3.24	-0.21 – 0.28	**4.7x 10^{-5}**	0.749	10.51
DHEA-sulfate	-0.25	0.08	-0.27	0.09	-0.44 – -0.06	-0.47 – -0.07	**0.014**	**0.014**	8.43
Time*	0.89	0.09	0.64	0.02	0.71 – 1.08	0.59 – 0.68	**1.4x 10^{-20}**	**1.8x 10^{-119}**	1032.00
Spray [p]	-0.09	0.36	-0.34	0.09	-0.86 – 0.68	-0.54 – -0.15	0.807	**0.001**	23.82
DHEA-sulfate × Time*	-0.03	0.02	-0.03	0.02	-0.07 – 0.02	-0.07 – 0.02	0.244	0.244	1032.00
DHEA-sulfate × Spray [p]	-0.08	0.09	-0.08	0.09	-0.26 – 0.11	-0.28 – 0.11	0.397	0.389	20.04
Time* × Spray [p]	-0.19	0.13	-0.22	0.03	-0.45 – 0.07	-0.28 – -0.15	0.160	**1.1x 10^{-10}**	1032.00
(DHEA-sulfate × Time*) × Spray [p]	-0.02	0.03	-0.02	0.03	-0.08 – 0.04	-0.09 – 0.04	0.523	0.523	1032.00
**Random Effects**									
σ^2	0.42								
$\tau_{00\ id}$	0.22								
$\tau_{11\ id.order2}$	0.71								
$\tau_{11\ id.order3}$	0.26								
$\tau_{11\ id.order4}$	0.57								
ρ_{01}	0.03								
	-0.36								
	-0.43								
ICC	0.35								
N_{id}	11								
Observations	1075								
Marginal R^2 / Conditional R^2	0.482 / 0.662								
AIC	2318.019								

DHEA-sulfate: Dehydroepiandrostendione-sulfate

*centered (scaled)

	Change in glucose infusion rate, interaction model with SHBG								
Predictors	*Estimates*	*std. Error*	*std. Beta*	*standardized std. Error*	*CI*	*standardized CI*	*p*	*std. p*	*df*
(Intercept)	1.08	0.30	0.14	0.10	0.46 – 1.70	-0.08 – 0.37	**0.002**	0.196	12.34
SHBG	0.00	0.00	0.09	0.10	-0.00 – 0.01	-0.11 – 0.29	0.346	0.374	19.80
Time*	0.62	0.12	0.85	0.05	0.37 – 0.86	0.75 – 0.95	**1.4×10^{-6}**	**2.3×10^{-37}**	181.44
Spray [p]	-0.00	0.26	-0.25	0.09	-0.52 – 0.51	-0.43 – -0.08	0.986	**0.006**	69.27
SHBG × Time*	0.00	0.00	0.06	0.05	-0.00 – 0.01	-0.04 – 0.17	0.226	0.226	180.86
SHBG × Spray [p]	-0.00	0.00	-0.11	0.08	-0.01 – 0.00	-0.27 – 0.05	0.177	0.180	83.91
Time* × Spray [p]	-0.20	0.17	-0.27	0.07	-0.54 – 0.14	-0.41 – -0.13	0.247	**0.0003**	180.03
(SHBG × Time*) × Spray [p]	-0.00	0.00	-0.02	0.07	-0.01 – 0.00	-0.16 – 0.13	0.804	0.804	180.30
**Random Effects**									
σ^2	0.36								
$\tau_{00\ id}$	0.18								
$\tau_{11\ id.order2}$	0.19								
$\tau_{11\ id.order3}$	0.03								
$\tau_{11\ id.order4}$	0.13								
ρ_{01}	0.14								
	-0.18								
	-0.69								
N_{id}	11								
Observations	213								
Marginal R^2 / Conditional R^2	0.662 / NA								
AIC	524.219								

SHGB: Sexual-hormone binding globulin

*centered (scaled)

	Change in glucose infusion rate, interaction model with FSH								
Predictors	*Estimates*	*std. Error*	*std. Beta*	*standardized std. Error*	*CI*	*standardized CI*	*p*	*std. p*	*df*
(Intercept)	1.17	0.22	0.08	0.11	0.73 – 1.61	-0.16 – 0.31	**7x 10^{-6}**	0.484	15.48
FSH	0.03	0.04	0.03	0.07	-0.06 – 0.11	-0.10 – 0.16	0.549	0.645	57.54
Time*	0.54	0.12	0.84	0.05	0.31 – 0.77	0.74 – 0.94	**5.7x 10^{-6}**	**4.5x 10^{-37}**	176.84
Spray [p]	-0.06	0.25	-0.23	0.09	-0.58 – 0.45	-0.41 – -0.05	0.801	**0.012**	62.90
FSH × Time*	0.05	0.03	0.10	0.05	0.00 – 0.10	0.00 – 0.21	**0.049**	**0.049**	176.78
FSH × Spray [p]	-0.06	0.06	-0.08	0.09	-0.17 – 0.06	-0.26 – 0.10	0.335	0.371	55.53
Time* × Spray [p]	-0.08	0.16	-0.26	0.07	-0.39 – 0.23	-0.40 – -0.12	0.601	**0.0004**	177.50
(FSH × Time*) × Spray [p]	-0.04	0.04	-0.08	0.07	-0.11 – 0.03	-0.22 – 0.07	0.299	0.299	176.68
**Random Effects**									
σ^2	0.36								
$\tau_{00\ id}$	0.17								
$\tau_{11\ id.order2}$	0.28								
$\tau_{11\ id.order3}$	0.08								
$\tau_{11\ id.order4}$	0.13								
ρ_{01}	0.10								
	-0.43								
	-0.57								
N_{id}	11								
Observations	213								
Marginal R^2 / Conditional R^2	0.666 / NA								
AIC	500.646								

FSH: Follicle-stimulating hormone

*centered (scaled)

	Change in glucose infusion rate, interaction model with LH								
Predictors	*Estimates*	*std. Error*	*std. Beta*	*standardized std. Error*	*CI*	*standardized CI*	*p*	*std. p*	*df*
(Intercept)	1.17	0.14	0.07	0.09	0.87 – 1.48	-0.14 – 0.27	**$4.4x\ 10^{-7}$**	0.490	10.19
LH	0.03	0.04	0.04	0.06	-0.05 – 0.11	-0.08 – 0.17	0.414	0.504	57.42
Time*	0.62	0.08	0.84	0.05	0.46 – 0.77	0.74 – 0.94	**$3.2x\ 10^{-13}$**	**$9.2x\ 10^{-38}$**	178.12
Spray [p]	0.01	0.18	-0.29	0.09	-0.35 – 0.36	-0.47 – -0.11	0.977	**0.002**	78.69
LH × Time*	0.05	0.02	0.10	0.05	0.00 – 0.10	0.01 – 0.20	**0.039**	**0.039**	184.31
LH × Spray [p]	-0.15	0.05	-0.21	0.09	-0.25 – -0.04	-0.39 – -0.04	**0.009**	**0.015**	97.79
Time* × Spray [p]	-0.03	0.11	-0.29	0.07	-0.24 – 0.19	-0.43 – -0.15	0.813	**$7.6x\ 10^{-5}$**	181.21
(LH × Time*) × Spray [p]	-0.09	0.04	-0.19	0.07	-0.16 – -0.02	-0.33 – -0.04	**0.011**	**0.011**	182.91
**Random Effects**									
σ^2	0.35								
$\tau_{00\ id}$	0.17								
$\tau_{11\ id.order2}$	0.35								
$\tau_{11\ id.order3}$	0.12								
$\tau_{11\ id.order4}$	0.17								
ρ_{01}	-0.00								
	-0.82								
	-0.41								
N_{id}	11								
Observations	213								
Marginal R^2 / Conditional R^2	0.680 / NA								
AIC	495.657								

LH: Luteinizing hormone

*centered (scaled)

	Change in glucose infusion rate, interaction model with AMH								
Predictors	*Estimates*	*std. Error*	*std. Beta*	*standardized std. Error*	*CI*	*standardized CI*	*p*	*std. p*	*df*
(Intercept)	2.59	0.27	0.11	0.12	2.01 – 3.17	-0.17 – 0.39	**9.2x 10^{-8}**	0.396	9.47
AMH	-0.33	0.08	-0.46	0.10	-0.49 – -0.17	-0.69 – -0.24	**0.001**	**0.0005**	15.27
Time*	1.02	0.06	0.63	0.02	0.91 – 1.13	0.59 – 0.68	**2.2x 10^{-66}**	**1.6x 10^{-122}**	1032.00
Spray [p]	-1.19	0.15	-0.54	0.06	-1.51 – -0.87	-0.67 – -0.42	**2x 10^{-6}**	**2.4x 10^{-6}**	9.83
AMH × Time*	-0.08	0.02	-0.10	0.02	-0.11 – -0.05	-0.14 – -0.06	**8.1x 10^{-7}**	**8.1x 10^{-7}**	1032.00
AMH × Spray [p]	0.18	0.04	0.25	0.06	0.09 – 0.27	0.13 – 0.37	**0.001**	**0.001**	11.51
Time* × Spray [p]	-0.23	0.09	-0.21	0.03	-0.40 – -0.05	-0.28 – -0.15	**0.010**	**1x 10^{-10}**	1032.00
(AMH × Time*) × Spray [p]	-0.01	0.03	-0.02	0.03	-0.06 – 0.04	-0.08 – 0.05	0.617	0.617	1032.00
**Random Effects**									
σ^2	0.41								
$\tau_{00\ id}$	0.27								
$\tau_{11\ id.order2}$	0.54								
$\tau_{11\ id.order3}$	0.10								
$\tau_{11\ id.order4}$	0.93								
ρ_{01}	-0.42								
	0.04								
	-0.15								
ICC	0.40								
N_{id}	11								
Observations	1075								
Marginal R^2 / Conditional R^2	0.527 / 0.715								
AIC	2278.580								

AMH: Anti-Müllerian hormone

*centered (scaled)

Supplementary Material S1: Reproducibility and reliability of cerebral blood flow measures

Reproducibility and reliability of global and hypothalamus cerebral blood flow was characterized in 110 healthy individuals (49.1% women, 46.4% overweight and obese, age range 21 – 74 years, BMI range 18 – 49 kg/m^2) with available cerebral blood flow measurements as in the current study (Kullmann et al., 2015; Wagner et al., 2023). Participants were enrolled in studies from 2014 to 2018 with two measurement time points separated by 2 to 6 weeks without a lifestyle or pharmaceutical Spray in between. CBF measurement acquired in the fasted state prior to intranasal application of insulin or placebo were analyzed. Reproducibility was characterized using the within-subject coefficient of variation (CV). Reliability was measured using a two-way mixed model intraclass correlation coefficient (ICC range: 0-1, values >0.75 are classified as excellent reliability (Shrout and Fleiss, 1979). There was no significant difference between measurement days for global and hypothalamus CBF (p>0.05, paired t-test). Reproducibility and reliability were high, indicated by low CV (CV_{global}= 0.06±0.05, $CV_{hypothalamus}$= 0.12±0.09) and high ICC values (ICC_{global}= 0.904, $ICC_{hypothalamus}$= 0.770).

Supplementary Material S2: Effect of intranasal placebo on cerebral blood flow

We investigated the effect of intranasal placebo from before to 30 min after application on regional cerebral blood flow of available CBF data (*N*=110) based on previous studies (Kullmann et al., 2015; Wagner et al., 2023). We identified an increase in CBF 30 min post application in left occipital cortex (PFWE<0.05, corrected for multiple comparisons; MNI coordinate: x -21, y -97, z 2). Furthermore, we extracted regional CBF of the hypothalamus before and 30 min after placebo administration of the 110 participants. No significant change in hypothalamic blood flow was observed in response to placebo nasal spray (mean±SD: Hypothalamus CBF_{pre} 35.7±8.7 ml/100g /min; Hypothalamus $CBF_{placebo}$ 36.6±9.5 ml/100g /min, p>0.05).

References

Kullmann, S., Heni, M., Veit, R., Scheffler, K., Machann, J., Häring, H.-U., Fritsche, A., Preissl, H., 2015. Selective insulin resistance in homeostatic and cognitive control brain areas in overweight and obese adults. Diabetes Care 38, 1044–1050. https://doi.org/10.2337/dc14-2319

Shrout, P.E., Fleiss, J.L., 1979. Intraclass correlations: uses in assessing rater reliability. Psychol Bull 86, 420–428. https://doi.org/10.1037//0033-2909.86.2.420

Wagner, L., Veit, R., Kübler, C., Fritsche, A., Häring, H.-U., Birkenfeld, A.L., Heni, M., Preissl, H., Kullmann, S., 2023. Brain insulin responsiveness is linked to age and peripheral insulin sensitivity. Diabetes Obes Metab. https://doi.org/10.1111/dom.15094
