# Supplementary material for: Brain insulin action on peripheral insulin sensitivity in women depends on menstrual cycle phase
Source: Nat Metab. 2023 Sep 21;5(9):1475–82. doi: 10.1038/s42255-023-00869-w (PMC10513929; doi:10.1038/s42255-023-00869-w)
Supplement: Supplementary file 2 — Reporting Summary [file 42255_2023_869_MOESM2_ESM.pdf]

nature portfolio

Corresponding author(s): Martin Heni

Last updated by author(s): Jul 5, 2023

Reporting Summary

Nature Portfolio wishes to improve the reproducibility of the work that we publish. This form provides structure for consistency and transparency in reporting. For further information on Nature Portfolio policies, see our Editorial Policies and the Editorial Policy Checklist.

Statistics

For all statistical analyses, confirm that the following items are present in the figure legend, table legend, main text, or Methods section.

n/a	Confirmed	
☐	☒	The exact sample size (*n*) for each experimental group/condition, given as a discrete number and unit of measurement
☐	☒	A statement on whether measurements were taken from distinct samples or whether the same sample was measured repeatedly
☐	☒	The statistical test(s) used AND whether they are one- or two-sided<br>*Only common tests should be described solely by name; describe more complex techniques in the Methods section.*
☐	☒	A description of all covariates tested
☒	☐	A description of any assumptions or corrections, such as tests of normality and adjustment for multiple comparisons
☐	☒	A full description of the statistical parameters including central tendency (e.g. means) or other basic estimates (e.g. regression coefficient) AND variation (e.g. standard deviation) or associated estimates of uncertainty (e.g. confidence intervals)
☐	☒	For null hypothesis testing, the test statistic (e.g. *F*, *t*, *r*) with confidence intervals, effect sizes, degrees of freedom and *P* value noted<br>*Give P values as exact values whenever suitable.*
☒	☐	For Bayesian analysis, information on the choice of priors and Markov chain Monte Carlo settings
☒	☐	For hierarchical and complex designs, identification of the appropriate level for tests and full reporting of outcomes
☒	☐	Estimates of effect sizes (e.g. Cohen's *d*, Pearson's *r*), indicating how they were calculated

Our web collection on statistics for biologists contains articles on many of the points above.

Software and code

Policy information about availability of computer code

Data collection	none
Data analysis	R (version 4.2.1). R packages: lme4 library (Version 1.1-31); lmerTest library (version 3.1-3). G*power (version 3.1.9.3). ASLtbx with SPM12 (Wellcome Trust Centre for Neuroimaging)

For manuscripts utilizing custom algorithms or software that are central to the research but not yet described in published literature, software must be made available to editors and reviewers. We strongly encourage code deposition in a community repository (e.g. GitHub). See the Nature Portfolio guidelines for submitting code & software for further information.

Data

Policy information about availability of data

All manuscripts must include a data availability statement. This statement should provide the following information, where applicable:

- Accession codes, unique identifiers, or web links for publicly available datasets
- A description of any restrictions on data availability
- For clinical datasets or third party data, please ensure that the statement adheres to our policy

The data generated during the current study are shared with researches upon reasonable request. Requests will be promptly reviewed by the Data Access Steering Committee of the Institute for Diabetes Research and Metabolic Diseases, Tübingen, Germany. Any data and materials that can be shared will be released via a Material Transfer Agreement.

Human research participants

Policy information about studies involving human research participants and Sex and Gender in Research.

Reporting on sex and gender	In our previous work, we found that brain insulin action modulates peripheral insulin sensitivity in lean men (Heni et al., 2014). We now studied metabolic (clamp study group) and hypothalamic effects (fMRI study group) of insulin delivery to the brain in young, lean naturally-cycling women. The biological sex of the participants was ensured by the sex hormone analysis performed to determine the menstrual cycle phase.
Population characteristics	We studied young, lean, healthy, naturally cycling women. This is presented as table 1 in the manuscript.
Recruitment	Participants were recruited by University-wide email anouncements. Therefore, participants with high levels of education and a high socio-economic status are enriched in this study population. Given the placebo-controlled cross-over design, we do not expect an impact on the results.
Ethics oversight	The study (NCT03929419) received approval by the local ethics committee (Ethics Committee of the Medical Faculty of the Eberhard Karls University and the University Hospital Tübingen) and was conducted according to the relevant guidelines and regulations.

Note that full information on the approval of the study protocol must also be provided in the manuscript.

Field-specific reporting

Please select the one below that is the best fit for your research. If you are not sure, read the appropriate sections before making your selection.

☒ Life sciences ☐ Behavioural & social sciences ☐ Ecological, evolutionary & environmental sciences

For a reference copy of the document with all sections, see nature.com/documents/nr-reporting-summary-flat.pdf

Life sciences study design

All studies must disclose on these points even when the disclosure is negative.

Sample size	For the clamp study, a sample size of n=10 was calculated (alpha = 0.05, power(1- ß error prob)=0.9, G*power) based on an effect size of Cohens d=1.178 , which we have achieved in our previous study investigation glucose infusion rates in response to intranasal insulin in lean men (Heni et al., 2014). To compensate for potential drop outs, n=2 were additionally recruited.
Data exclusions	We excluded one participant from the analysis of the clamp study and one from the fMRI study, since the timepoint of examination did not meet the allocated cycle phase. This is presented in the supplementary figure 1&2 of the manuscript.
Replication	We confirmed the findings from the clamp study group in another group of participants using fMRI.
Randomization	Participants received insulin and placebo spray during the hyperinsulinemic euglycemic clamps in a randomized order. The initial cycle phase for the experiments was chosen in a random order. To further exclude any order effects, we have included "order" in the mixed models as random slope. For the fMRI study, solely one experimanetal condition with intranasal insulin was tested (no placebo condition included), therefore random allocation to experimental groups is not applicable here.
Blinding	Clamp study (study group 1): We performed two clamp experiments per cycle phase using a single-blind cross-over design. Hence, only study participants were blinded for the type of spray (insulin or placebo), while study personnel were not blinded during data analysis and data collection due to safety reasons.<br>fMRI Study (study group 2): Blinding was not relevant for the fMRI study, since solely insulin spray (without a placebo control) was used.

Reporting for specific materials, systems and methods

We require information from authors about some types of materials, experimental systems and methods used in many studies. Here, indicate whether each material, system or method listed is relevant to your study. If you are not sure if a list item applies to your research, read the appropriate section before selecting a response.

Materials & experimental systems

n/a	Involved in the study
☒	☐ Antibodies
☒	☐ Eukaryotic cell lines
☒	☐ Palaeontology and archaeology
☒	☐ Animals and other organisms
☐	☒ Clinical data
☒	☐ Dual use research of concern

Methods

n/a	Involved in the study
☒	☐ ChIP-seq
☒	☐ Flow cytometry
☐	☒ MRI-based neuroimaging

Clinical data

Policy information about clinical studies

All manuscripts should comply with the ICMJE guidelines for publication of clinical research and a completed CONSORT checklist must be included with all submissions.

Clinical trial registration: NCT03929419

Study protocol: We have enclosed the study protocol for the review process of nature metabolism. The protocol will moreover included in this publication as supplemental file.

Data collection: Data were collected at the University Hospital of Tübingen between April 2019 and March 2021.

Outcomes: Primary outcome was the change in glucose infusion rate after nasal spray administration during the hyperinsulinemic euglycemic clamp experiment.

Secondary Outcome Measures:

Differential effects dependent on female sexual hormones. Tested as correlation of the change in peripheral insulin sensitivity by central insulin action dependent on the time point in menstrual cycle.

Effect of menstrual cycle on insulin sensitivity before spray application assessed by hyperinsulinemic euglycemic clamp.

Effect of menstrual cycle on brain insulin sensitivity:
Hypothalamic insulin sensitivity was assessed by functional magnetic resonance imaging combined with intranasal insulin administration during the follicular and the luteal phase of the menstrual cycle.

Magnetic resonance imaging

Experimental design

Design type: Resting state

Design specifications: Resting-state cerebral blood flow measurements before and 30 min after intranasal insulin.

Behavioral performance measures: We do not report behavioral performance measures in this manuscript.

Acquisition

Imaging type(s): Functional

Field strength: 3 Tesla

Sequence & imaging parameters: Arterial spin labeling (PASL) images were obtained with a PICORE-Q2TIPS (proximal inversion with control for off-resonance effects (quantitative imaging of perfusion by using a single subtraction) sequence by using a frequency offset corrected inversion pulse and echo planar imaging readout for acquisition. In addition, high-resolution T1-weighted anatomical images were obtained.

Area of acquisition: Whole-brain

Diffusion MRI: ☐ Used ☒ Not used

Preprocessing

Preprocessing software: Image preprocessing was performed by using the ASLtbx with SPM12 (Wellcome Trust Centre for Neuroimaging). Functional images were motion corrected, coregistered to the individual anatomical image and smoothed (full width at half maximum: 6 mm).

Normalization	The high resolution T1-weighted image was normalized in Montreal Neurological Institute space (1 x 1 x 1 mm) using SPM12's unified segmentation normalization, and the resulting parameter file was used with the individual coregistered CBF maps in normalized space (3 x 3 x 3 mm).
Normalization template	normalized in Montreal Neurological Institute (MNI305)
Noise and artifact removal	Six head motion parameters. No participant had head motion with more than 2.0 mm maximum displacement or 2.0° of any angular motion.
Volume censoring	NA

Statistical modeling & inference

Model type and settings	NA
Effect(s) tested	Hypothalamic cerebral blood flow before and after nasal insulin administration was compared by paired t-tests (two tailed) for the follicular and luteal phase separately.

Specify type of analysis: ☐ Whole brain ☒ ROI-based ☐ Both

Anatomical location(s)	CBF was extracted from the hypothalamus as our region of interest, based on previous findings (Kullmann et al 2015, Diab Care)
Statistic type for inference (See Eklund et al. 2016)	NA
Correction	NA

Models & analysis

n/a	Involved in the study
☒	☐ Functional and/or effective connectivity
☒	☐ Graph analysis
☒	☐ Multivariate modeling or predictive analysis
