# Supplementary material for: Brain insulin action on peripheral insulin sensitivity in women depends on menstrual cycle phase
Source: Nat Metab. 2023 Sep 21;5(9):1475–82. doi: 10.1038/s42255-023-00869-w (PMC10513929; doi:10.1038/s42255-023-00869-w)
Supplement: Supplementary file 3 — Translated final study protocol of the trial. [file 42255_2023_869_MOESM3_ESM.pdf]

Study protocol

for the study

„Influence of insulin action in the CNS on insulin sensitivity of various peripheral organs in women“

15.01.2020 Version 3.1

Head of the study and contact person

Prof. Dr. med. Martin Heni (PI)<br>Medical clinic IV<br>University of Tübingen<br>Otfried-Müller-Str. 10<br>72076 Tübingen<br>Germany<br>Tel. 07071/29-80344; Fax 07071/29-5977	Prof. Dr. med. Andreas Fritsche<br>Medical clinic IV<br>University of Tübingen<br>Otfried-Müller-Str. 10<br>72076 Tübingen<br>Germany<br>Tel. 07071/29-80590, Fax: 07071/29-5277

Investigators

Prof. Dr. med. Martin Heni
Medical clinic IV, University Tübingen
PD Dr. med. Robert Wagner
Medical clinic IV, University Tübingen
Dr. med. Caroline Willmann
Medical clinic IV, University Tübingen
Dr. med. Charlotte Benkendorff
Medical clinic IV, University Tübingen

Funding

Funding through the German Center for Diabetes Research (DZD) e.V.

Declaration of consent from the head of the institute

The signatories read the study protocol and agrees with its content.

Institute director	______________________<br>Prof. A. L. Birkenfeld	______________________<br>Date

1. Background

Pathogenesis of type 2 diabetes mellitus

Type 2 diabetes mellitus is a disease with a significant and increasing prevalence (age of 50-80 years: 5-18%) and is associated with serious macro- and microangiopathic complications. The heterogeneous clinical picture of type 2 diabetes mellitus is caused by genetic and environmental factors [1].

Two important mechanisms play a role in the pathogenesis: on the one hand, the insulin resistance of the target tissue and, on the other hand, a secretion defect of the β-cells of the islet organ of the pancreas [2–4].

One of the main effects of insulin in target tissues is to control the uptake of glucose into cells. The liver, skeletal muscles and adipose tissue are considered to be insulin-sensitive target tissues. Insulin resistance of these tissues is considered to be one of the main defects in the development of type 2 diabetes mellitus. To quantify insulin sensitivity or resistance, the effect of insulin on glucose uptake is usually measured in a hyperinsulinemic-euglycemic clamp [5].

Insulin action in the CNS

The uptake of glucose into neurons occurs via an insulin-independent mechanism, so that the CNS does not depend on the presence of insulin for glucose metabolism. Nevertheless, insulin receptors are present in the CNS [6]. The specific insulin action in the CNS became the focus of research when the insulin receptor in animals was selectively knocked out in the brain: the test animals consumed much more food than controls, became overweight and developed insulin resistance throughout the body [7]. In further experiments, various functions of central insulin action in animals were discovered. Insulin action in the brain influences e.g. food intake, energy expenditure, systemic lipolysis, systemic glucose metabolism, systemic insulin sensitivity and insulin secretion from the beta cells [8].

In humans, we and others have also been able to show that insulin has an effect in the brain:

Own results in this area

Using magnetoencephalography (MEG), we were able to demonstrate insulin effects on the cerebral cortex in humans. The insulin effects we have detected are clearly correlated with the weight of the people examined [9]. In addition, the effect of central nervous insulin

decreases with increased saturated free fatty acids in the plasma [10] and with increasing age [11]. Impaired transport across the blood-brain barrier also seems to play a pathogenetic role in brain insulin resistance [12].

The intranasal administration of insulin allows insulin to be transported selectively to the human brain, bypassing the blood-brain barrier [13, 14]. Using this method, we were able to show that nasal insulin primarily affects the neuronal response to visual food stimuli [15]. We were also able to show that nasal insulin has no effect on global cerebral blood flow [16]. Even after nasal insulin application, the higher the weight, the lower the insulin effect [17, 18]. Thus, we were able to confirm results obtained with intravenous insulin with intranasal insulin.

To date, there have only been a few experimental studies in humans on the question of how insulin action in the brain affects peripheral metabolism. In cooperation with colleagues in Lübeck and New York, we were able to show that intranasal administration of insulin systemically suppresses lipolysis [19]. In a further study, there were initial indications that regulation of peripheral insulin sensitivity through insulin action in the central nervous system is also possible in humans, as shown in laboratory animals [20]. We examined this indirect result in more detail in another study. We measured peripheral insulin sensitivity in healthy men using the hyperinsulinemic-euglycemic clamp. During the clamp we applied 160 U insulin or placebo intranasally. While insulin sensitivity did not change after placebo spray, there was a significant improvement in peripheral insulin sensitivity after insulin nasal spray.

Whether these results can also be transferred to women cannot be answered with the previous experiment. The group of Hallschmid et al. [21] was able to show that there are gender-specific differences in the response to intranasal insulin. In this study, men and women applied intranasal insulin for 8 weeks. The men showed a reduction in weight, which was not present in the women. The female sex hormones thus appear to have an influence on cerebral insulin action. Since the female sex hormones are subject to cycle-related fluctuations and it has long been suspected that this affects insulin sensitivity, women without hormonal contraception should be examined in the first and second half of the cycle.

For this purpose, the now planned study is to be carried out.

2. Research question

We hypothesize insulin action in the central nervous improves peripheral insulin sensitivity in women and that this improvement correlates with changes in autonomic and central nervous system activity. In addition, it should be investigated whether there are cycle-dependent differences.

3. Study population

A total of 10 subjects (normal weight, BMI up to 24 kg/m², no hormonal contraceptives) will be included. In previous studies with nasal insulin, there were clear sex differences. In order to be able to compare the collected data with existing data from men [22], similar inclusion criteria should be used. In addition, peripheral insulin sensitivity is significantly influenced by the menstrual cycle [23]. We will therefore examine the subjects in the first and second half of the cycle. The study participants are recruited by specifically addressing suitable participants and by advertising via circular mails and notices.

<u>Inclusion Criteria:</u>

female adult volunteers

HbA1c <6.0%

Age between 18 and 30 years

Standard routine laboratory

No underlying diseases

No medication intake

No hormonal contraception

Understanding of declaration of consent, study explanations and instructions

<u>Exclusion criteria</u>

• People who have non-removable metal parts in or on their body, such as:

pacemaker
artificial heart valves
metal prostheses
implanted magnetic metal parts (screws, plates from surgeries)
coil
Metal shards/shrapnel

fixed braces
Retainers go over more than four teeth
acupuncture needle
insulin pump
intraport
tattoos, eye shadow etc.

• People with reduced temperature sensitivity and/or increased sensitivity towards warming of the body
• Cardiovascular disease cannot be ruled out, such as manifest coronary heart disease, heart failure greater than NYHA 2, previous heart attack, condition after a stroke
• People with a hearing disorder or an increased sensitivity to loud noises
• Persons with claustrophobia (claustrophobia)
• Subjects who are minors or who are unable to consent are also excluded
• Pregnancy or lactation
• Subjects who had an operation less than 3 months ago
• Concurrent participation in other interventional studies
• Acute illness or infection within the last 4 weeks
• Neurological and psychiatric disorders
• Subjects with hemoglobin values Hb<12g/dl (at screening)
• Allergic diseases
• Individuals with a history of heparin-induced thrombocytopenia (HIT).

Subjects must be in good general condition with no serious underlying diseases assessed during the clinical examination by the investigator. If there are doubts about these requirements, further laboratory values may be determined after a clinical examination.

4. Sample size calculation

In the previous study [22], the glucose infusion rate in lean men was influenced by insulin nasal spray during the clamp. The effect size was (Cohen's) dz = 1.178. Based on this information we calculated the sample size (alpha = 0.05, power (1-beta error probability) = 0.9). A sample size of n = 10 is required. If there are drop-outs, these subjects are replaced by new ones who have to go through the experiment from the beginning.

5. Study course and research methods

At least one day before the start of the study, the participants will be informed about the study. Participants can only take part in the study after they have given their written consent. At the first appointment (metabolic characterization, precise examination of exclusion criteria), the subjects are clinically examined after anamnesis, including determination of laboratory values such as blood count, inflammation values, electrolytes, kidney values, liver values, lipids, coagulation, HbA1c, hormones, urine tests and anthropometric measurements. In addition, a detailed cycle anamnesis takes place. In addition, DNA for genotyping for metabolically relevant polymorphisms is optionally preserved with the separate consent of the subjects (project number genetic biobank 175/2017BO1).

The participants will be examined on two independent days in the first and second half of the cycle using a hyperinsulinemic euglycemic clamp and functional magnetic resonance imaging (fMRI), once with nasal insulin administration, once with nasal placebo administration. The order of investigations (nasal insulin, nasal placebo) is randomized and blinded, the interval between trials will be 2 days to 8 weeks.

Euglycemic hyperinsulinemic clamp

The investigation is carried out after an overnight fasting period in a lying position. A venous access is placed in an elbow vein for the infusion of glucose (10%) and insulin. Another venous access is placed in a vein on the back of the hand. This hand is warmed with the help of heating pads (up to a maximum of 40°C), which allows the collection of arterialized venous blood (continuous determination of glucose, further blood collection [labelled glucose, insulin, C-peptide, free fatty acids, nitroso-glutathione, as well as blood for preservation for later investigations of other hormones and metabolites]).

After taking the first blood samples, the continuous infusion of [6,6-$^2H_2$] glucose is started. This starts with a bolus of 3.6 mg/kg followed by a continuous infusion of 0.036 mg/kg/min. After 2 hours, an insulin infusion (human insulin in 0.9% NaCl solution) is started. After an insulin bolus, 0.25 mU/kg/min of insulin is infused continuously for 210 minutes. During the entire duration of the insulin infusion, the blood sugar is measured every 5 - 10 min and kept in the normal range by means of a variably infused 20% glucose solution with labeled [6,6-$^2H_2$] glucose. Before and twice after nasal insulin/placebo administration at minute 90 of the

clamp, insulin sensitivity is calculated as an index from measured insulin levels and glucose infusion rate. Endogenous glucose production (EGP) will be calculated for the same time periods using Steele's equation. From these calculations, the insulin sensitivity of the liver, muscles and other tissues can be estimated. The respective blood samples are given in the following overview. The total volume of blood samples is approximately 130 ml per test.

In the experiment, insulin levels in the central nervous system will be increased 90 minutes after the onset of the hyperinsulinemic euglycemic clamp via an intranasal administration. For this purpose, human insulin is used in the form of an intranasal spray (16 strokes of 10 U), with a corresponding amount of placebo being used in the placebo test. On the placebo days, the *iv* insulin infusion was slightly increased for 20 minutes after administration of the nasal spray in order to simulate spill-over effects from the insulin days.

During the times when insulin sensitivity is calculated, we will record an ECG for about 10 minutes to record heart rate variability as a measure of the activity of the autonomic nervous system.

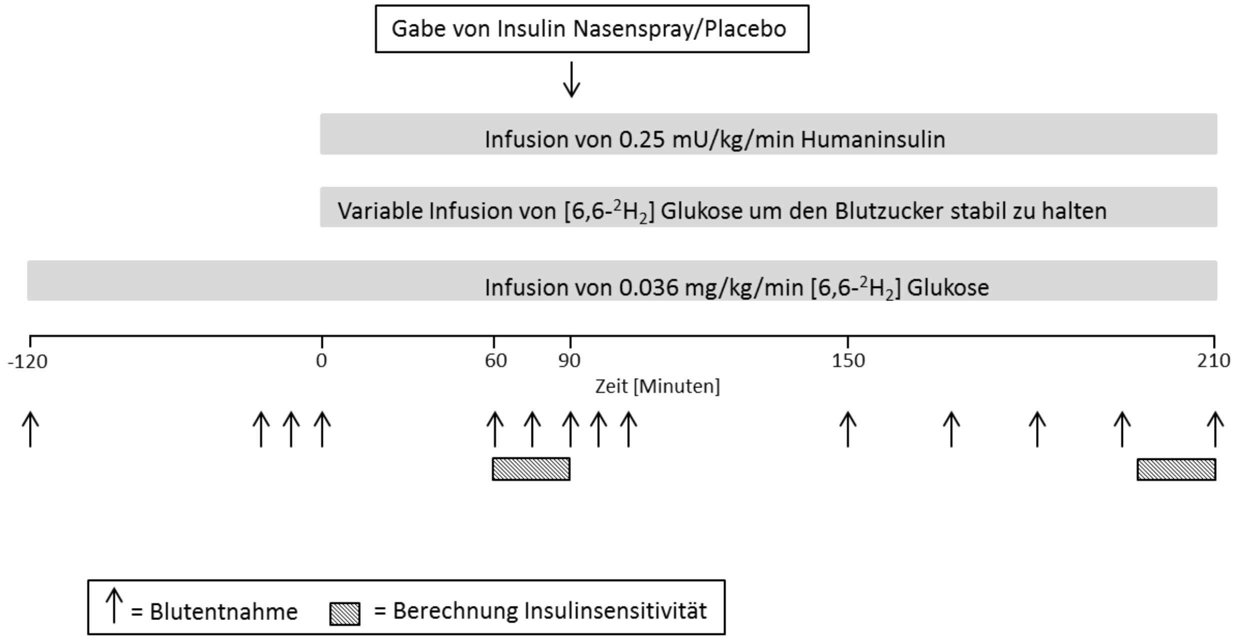

Abb. 1. Test procedure: Clamp

Functional MRI measurement

The participants will be subjected to fMRI measurement and behavioral tests on two independent days (first and second half of the cycle). Measurements will start at 8 a.m. on both days for all subjects in order to rule out circadian fluctuations. Intranasal insulin is administered on both examination days.

The neurophysiological measurements on the MRI scanner are made up of two measurement blocks each. The first part always takes place basally, with the second being carried out after the intranasal administration of insulin. The breaks between the measurements are used to fill out questionnaires on eating behavior and mood. The fMRI measurements each last about 40 minutes, with the test persons having no task during the measurements in the basal measurement. An ECG is recorded during the MRI measurement.

During the first MRI measurement, an anatomical image of the brain is taken and a measurement is performed to quantitatively record the proton density (quantitative water content imaging). During the first and second fMRI measurement, the brain activity is measured at rest. The absolute cerebral blood flow (CBF) enables brain activity to be recorded in physiologically defined and thus comparable units (ml/100g/min) without stimulation. To capture coherent patterns of spontaneous blood oxygen level dependent (BOLD) fluctuations in the resting state, a standard fMRI BOLD sequence is performed directly after the CBF measurements. Furthermore, at the first and second measurement, a spectroscopic examination (1H-MRS) of the gray and white matter is recorded to detect metabolites that are modulated by inflammatory processes and intranasal insulin. Only in the second part of the fMRI measurement, subjects are asked to look at the food images and to press a button when a cross appears. This ensures their attention.

The examinations at the MRI are carried out by people with magnetic resonance imaging certification, at least 2 people are on site for each measurement. The examiners have extensive experience with the examination methods to be used.

Between the fMRI measurements, the participant`s subjective condition is checked using a visual analogue scale (eating, drinking, etc.). Mood is recorded using the German form of the positive and negative affect schedule (PANAS) [24]. Other questionnaires on eating behavior

include the Questionnaire on Eating Behavior [25] and the German version of the Food Cravings Questionaire [26]. Depression is recorded using the Beck Depression Inventory (BDI-II) [27].

Behavioral Tests:

Memory and attention tests:

After the fMRI experiment, the subjects will take part in behavioral tests after a 15-minute break. During the break, subjects are given a glass of water to drink. Then the food-relevant images from the second part of the fMRI measurement are presented to the subjects on a laptop. Subjects are asked to recall whether or not they have seen the images before (40% of the images are unknown) and then indicate how certain they are on a scale. Furthermore, the participants should rate how strongly appetite stimulating the pictures are.
After a short break, further behavioral tests are carried out which record the participant`s attention (trail-making test A&B) and memory (Hopkins-verbal learning test-revised).

Insulin in the brain acts on the dopamine system, this can be assessed through the learning task for approach and avoidance behaviors for rewards and punishments [28].

6. Compensation

Each subject receives an appropriate expense allowance of €500 for their participation in the study.

7. Risks for the participants and risk-benefit analysis

Risks:

Blood will be drawn from the participants several times during the study. This is done via a venous access that is placed in a vein on the back of the hand. In addition, a venous access is placed in the cubital vein. The puncture can be slightly painful and rarely lead to local irritation, bleeding, bruising and inflammation of the blood vessels and to incorrect puncture of other vessels and nerves. Localized infections around the puncture sites of the needles or cannulas as well as infections that spread to the whole body cannot be completely ruled out.

Side effects of elevated insulin levels (hyperinsulinemic euglycemic clamp) may include increased blood flow and occasionally dizziness. This is prevented by an additional infusion of full electrolyte solution (Jonosteril). A theoretically possible hypoglycaemia (blood sugar level below 50 mg/dl) is prevented by measuring glucose every 5-10 minutes and adjusting the glucose infusion rate. After the examination, any after-effects of the increased insulin levels with a possible drop in blood glucose into the hypoglycaemic range are counteracted by a carbohydrate-rich meal (minimum 50 g carbohydrates).

The infused glucose can lead to venous irritation, which is prevented by infusing the Jonosteril solution at the same time.

Human nasal insulin (Actrapid®)

Manufacturing and procurement

The commercially available insulin preparation Actrapid® in the form of an intranasal spray is used for the study. The manufacturer is the pharmaceutical company Novo Nordisk A/S Novo Allé, DK-2880 Bagsværd, Denmark.

The test preparation is filled into pump spray bottles and not modified.

Permit

Actrapid® is approved for the treatment of diabetes mellitus.

The dates of approval/renewal of approval for Actrapid® are: 09/2007

The insulin is not approved for intranasal application.

<u>Description/Identity</u>

Trade name:	Actrapid®
INN:	human insulin
Chemical Name:	Protein identical to human insulin, produced by appropriate purification or by genetic engineering
Excipients:	zinc chloride
	glycerol
	m-cresol
	Sodium hydroxide and/or hydrochloric acid (to adjust pH)
	water for injections

Placebo

Since this insulin solution to be administered intranasally is characterized by a characteristic odour, the placebo solution must have similar properties so that the two conditions are indistinguishable for the subjects. The insulin dilution solution (placebo) contains the following substances:

1 ml of the placebo solution contains:	2.7 mg of m-cresol
	16 mg glycerol
	water for injections

Side effects of Actrapid® nasal spray

In this study, Actrapid® is administered intranasally from a spray. The intranasal administration of peptides represents a mode of administration in which there is no significant increase in the peptide in the bloodstream, therefore no usual systemic side effects of insulin are to be expected.

There may be temporary symptoms in the nasal mucosa, such as local irritation, hypersensitivity and a feeling of dryness.

As with any medication, there is a possibility of allergic reactions. Since the examinations with insulin nasal spray take place at the University Hospital in Tübingen, the appropriate emergency utensils and procedures are available at all times.

For prescribing information on Insulin Actrapid® see attachment.

Side effects of placebo nasal spray

There may be temporary symptoms in the nasal mucosa, such as local irritation, hypersensitivity and a feeling of dryness.

Expected benefit:

Better understanding of how insulin action in the brain influences insulin sensitivity in the body in women and which central nervous centres and peripheral organs are involved. Also new insights into how the menstrual cycle affects insulin action in the brain and peripheral insulin sensitivity.

As this study is expected to provide a significant increase in knowledge about the pathogenesis of whole-body insulin resistance and type 2 diabetes mellitus and other diseases, we believe that the expected benefits justify the overall low risks.

8. Individual study termination

Criteria for the individual termination of the study

The study participants can withdraw from the study at any time and without giving reasons without suffering any disadvantages. Participation in the study is strictly voluntary.

The investigator, principal investigator, and sponsor may withdraw individual study participants if their safety or the achievement of study objectives are at risk. The following reasons can be decisive here:

- Non-compliance with study conditions
- Occurrence of pregnancy
- Other relevant deviations from the study protocol

Procedure in the event of an individual study termination

Each individual study termination must be documented in the patient file. The following information must be provided:

- Date and time of the study termination and the study day
- Reason for dropping out of the study or the note that this could not be determined if the study participant withdraws without giving a reason.

Any study participant who withdraws from the study prematurely after inclusion in the study will be replaced by a test person of the same weight group. Every replacement subject goes through the study from the beginning like a "new" subject.

9. Study duration

2 years

10. Data protection

As part of the study "Influence of insulin action in the CNS on insulin sensitivity of various peripheral organs in women" personal data (names, date of birth, address, previous findings, study-related findings, etc.) are collected and processed.

The data is documented and archived pseudonymously in a protected electronic database to which only authorized employees, including doctoral students who are bound to confidentiality and data secrecy, have access. In order to check the correct transfer of the treatment data from the medical file to the encrypted study database, authorized persons (so-called monitors) are allowed to look at the personal subject data that is related to the study. All employees involved are bound to confidentiality.

The data collected as part of the study can also be used and processed for future research projects at the clinic or institute.

The processing and use of the pseudonymised data take place on survey forms and electronic data carriers, usually for a period of 20 years, insofar as the purpose of the study does not require a longer storage period e.g. when entering into a database and in long-term studies.

The information obtained in the course of this study can also be transmitted for scientific purposes to cooperation partners within the scope of the European General Data Protection Regulation and to cooperation partners outside the European Economic Area, i.e. in countries with a lower level of data protection (this also applies to the USA). In this case, the security of your data cannot be guaranteed.

The research results from the study are published anonymously in specialist journals or in scientific databases. When the research results are published, the identity of the study participants will not be disclosed. However, the on-site investigators can trace back the data to the person with the help of a list of subjects in the event of queries.

The subject can request information about his stored data at any time and has the right to have incorrect data corrected. The subject can also request at any time that his data gets deleted or made anonymous so that a reference to the person can no longer be established.

The head of the study (Prof. Dr. Martin Heni) is responsible for data processing and compliance with the statutory data protection regulations.

Complaints can be submitted to the data protection officer of the University Hospital in Tübingen or the state data protection officer of the state of Baden-Württemberg.

The express consent of the study participant by signing the declaration of consent to data protection is required for the collection, storage, use and disclosure of the data.

Contact information:
Data Protection Officer of the University Hospital Tübingen
Postal address: Calwerstraße 7/4, 72076 Tübingen, Tel. 07071 29-87667, e-mail: dsb@med.uni-tuebingen.de

State representative for data protection and freedom of information in Baden-Württemberg
Postal address: PO Box 10 29 32, 70025 Stuttgart
Tel.: 0711/615541-0, FAX: 0711/615541-15, email: poststelle@lfdi.bwl.de

If pathological findings are found during the examinations, the subjects will be informed of this. Only subjects who agree to this procedure can take part in the study.

Blood samples

Blood samples are stored in a lockable freezer for a maximum of 20 years. These may only be removed with the approval of the director of studies.

11. Ethical aspects

Declaration of Helsinki and GCP

This study is conducted in accordance with the ethical principles of the Declaration of Helsinki (revised version of 2002) and in accordance with the Good Clinical Practice Guideline of the International Conference on Harmonization (ICH-GCP-Guideline) as well as in accordance with the applicable legal requirements.

Storage of study documents

See data protection

12. Literature

1. Defronzo RA (2009) Banting Lecture. From the triumvirate to the ominous octet: a new paradigm for the treatment of type 2 diabetes mellitus. Diabetes 58:773–795

2. DeFronzo RA (2010) Insulin resistance, lipotoxicity, type 2 diabetes and atherosclerosis: the missing links. The Claude Bernard Lecture 2009. Diabetologia 53:1270–1287

3. Kahn SE (2003) The relative contributions of insulin resistance and beta-cell dysfunction to the pathophysiology of Type 2 diabetes. Diabetologia 46:3–19

4. Staiger H, Machicao F, Fritsche A, Häring H-U (2009) Pathomechanisms of type 2 diabetes genes. Endocr Rev 30:557–585

5. Ferrannini E, Mari A (1998) How to measure insulin sensitivity. J Hypertens 16:895–906

6. Morton GJ, Cummings DE, Baskin DG, Barsh GS, Schwartz MW (2006) Central nervous system control of food intake and body weight. Nature 443:289–295

7. Brüning JC, Gautam D, Burks DJ, Gillette J, Schubert M, Orban PC, Klein R, Krone W, Müller-Wieland D, Kahn CR (2000) Role of brain insulin receptor in control of body weight and reproduction. Science 289:2122–2125

8. Schwartz MW, Seeley RJ, Tschöp MH, Woods SC, Morton GJ, Myers MG, D'Alessio D (2013) Cooperation between brain and islet in glucose homeostasis and diabetes. Nature 503:59–66

9. Tschritter O, Preissl H, Hennige AM, et al (2006) The cerebrocortical response to hyperinsulinemia is reduced in overweight humans: A magnetoencephalographic study. Proc Natl Acad Sci 103:12103–12108

10. Tschritter O, Preissl H, Hennige AM, et al (2009) The Insulin Effect on Cerebrocortical Theta Activity Is Associated with Serum Concentrations of Saturated Nonesterified Fatty Acids. J Clin Endocrinol Metab 94:4600–4607

11. Tschritter O, Hennige AM, Preissl H, Grichisch Y, Kirchhoff K, Kantartzis K, Machicao F, Fritsche A, Häring H-U (2009) Insulin effects on beta and theta activity in the human brain are differentially affected by ageing. Diabetologia 52:169–171

12. Heni M, Schöpfer P, Peter A, Sartorius T, Fritsche A, Synofzik M, Häring H-U, Maetzler W, Hennige AM (2013) Evidence for altered transport of insulin across the blood-brain barrier in insulin-resistant humans. Acta Diabetol. https://doi.org/10.1007/s00592-013-0546-y

13. Born J, Lange T, Kern W, McGregor GP, Bickel U, Fehm HL (2002) Sniffing neuropeptides: a transnasal approach to the human brain. Nat Neurosci 5:514–516

14. Chapman CD, Frey WH 2nd, Craft S, Danielyan L, Hallschmid M, Schiöth HB, Benedict C (2012) Intranasal Treatment of Central Nervous System Dysfunction in Humans. Pharm Res. https://doi.org/10.1007/s11095-012-0915-1

15. Guthoff M, Grichisch Y, Canova C, Tschritter O, Veit R, Hallschmid M, Häring H-U, Preissl H, Hennige AM, Fritsche A (2010) Insulin modulates food-related activity in the central nervous system. J Clin Endocrinol Metab 95:748–755

16. Grichisch Y, Çavuşoğlu M, Preissl H, Uludağ K, Hallschmid M, Birbaumer N, Häring HU, Fritsche A, Veit R (2012) Differential effects of intranasal insulin and caffeine on cerebral blood flow. Hum Brain Mapp 33:280–287

17. Guthoff M, Stingl KT, Tschritter O, et al (2011) The insulin-mediated modulation of visually evoked magnetic fields is reduced in obese subjects. PloS One 6:e19482

18. Stingl KT, Kullmann S, Guthoff M, Heni M, Fritsche A, Preissl H (2010) Insulin modulation of magnetoencephalographic resting state dynamics in lean and obese subjects. Front Syst Neurosci 4:157

19. Iwen KA, Scherer T, Heni M, et al (2014) Intranasal insulin suppresses systemic but not subcutaneous lipolysis in healthy humans. J Clin Endocrinol Metab 99:E246-251

20. Heni M, Kullmann S, Ketterer C, et al (2012) Nasal insulin changes peripheral insulin sensitivity simultaneously with altered activity in homeostatic and reward-related human brain regions. Diabetologia 55:1773–1782

21. Hallschmid M, Benedict C, Schultes B, Fehm H-L, Born J, Kern W (2004) Intranasal insulin reduces body fat in men but not in women. Diabetes 53:3024–3029

22. Heni M, Wagner R, Kullmann S, et al (2014) Central insulin administration improves whole-body insulin sensitivity via hypothalamus and parasympathetic outputs in men. Diabetes 63:4083–4088

23. Yeung EH, Zhang C, Mumford SL, Ye A, Trevisan M, Chen L, Browne RW, Wactawski-Wende J, Schisterman EF (2010) Longitudinal Study of Insulin Resistance and Sex Hormones over the Menstrual Cycle: The BioCycle Study. J Clin Endocrinol Metab 95:5435–5442

24. Heinz Walter Krohne, Boris Egloff, Carl-Walter Kohlmann, Anja Tausch (1996) Untersuchungen mit einer deutsche Version der “Positive and Negative Affect Schedule” (PANAS). Diagnostica 42:139–156

25. Volker Pudel, Joachim Westenhöfer (1989) Fragebogen zum Eßverhalten (FEV). Handanweisung.

26. Meule A, Hermann T, Kübler A (2014) A short version of the Food Cravings Questionnaire—Trait: the FCQ-T-reduced. Front Psychol. https://doi.org/10.3389/fpsyg.2014.00190

27. Beck AT, Steer RA, Carbin MG (1988) Psychometric properties of the Beck Depression Inventory: Twenty-five years of evaluation. Clin Psychol Rev 8:77–100

28. Guitart-Masip M, Economides M, Huys QJM, Frank MJ, Chowdhury R, Duzel E, Dayan P, Dolan RJ (2014) Differential, but not opponent, effects of l-DOPA and citalopram on

action learning with reward and punishment. Psychopharmacology (Berl) 231:955–966
